# Peroxisomal membrane protein PMP70 confers drug resistance in colorectal cancer

**DOI:** 10.1038/s41419-025-07572-6

**Published:** 2025-04-14

**Authors:** Jinwen Yin, Yu Shao, Fengxing Huang, Yuntian Hong, Wanhui Wei, Congqing Jiang, Qiu Zhao, Lan Liu

**Affiliations:** 1 Department of Gastroenterology, Zhongnan Hospital of Wuhan University, Wuhan, 430000 China; 2 Hubei Clinical Center and Key Lab of Intestinal and Colorectal Diseases, Wuhan, 430000 China; 3 Liangzhu Laboratory, Zhejiang University, 1369 West Wenyi Road, Hangzhou, 311121 China; 4 Department of Colorectal and Anal Surgery, Zhongnan Hospital of Wuhan University, Wuhan, 430000 China; 5 Wuhan Clinical Research Center for Constipation and Pelvic Floor Disorders, Wuhan, 430000 China

**Keywords:** Gastrointestinal cancer, Chemotherapy, Peroxisomes

## Abstract

Metabolic reprogramming is a key contributor to cancer therapeutic resistance. Peroxisomes are highly metabolic organelles essential for lipid metabolism and reactive oxygen species (ROS) turnover. Recent studies pointed out that targeting peroxisomal genes could be a promising strategy for treating therapy-resistant cells. However, the role of peroxisomes in CRC chemoresistance remains largely unexplored. This study aimed to investigate the function of peroxisomes in CRC chemoresistance and uncover the underlying mechanisms. Our results showed that the protein level of peroxisome marker PMP70 was strongly correlated with oxaliplatin (LOHP)-treated tumor recurrence in CRC. LOHP was confirmed to induce pexophagy in CRC cells, whereas LOHP-resistant cells maintained stable peroxisome levels and resisted this selective autophagy. Moreover, depletion of PMP70 significantly reduced the viability of resistant CRC cells in response to LOHP, both in vitro and in vivo. Mechanistically, PMP70 acted as a potential protector against excessive lipid peroxidation (LPO) in PMP70^High^ and LOHP-resistant CRC cells. Additionally, PMP70-depleted cells exhibited an altered metabolic profile, characterized by reduced neutral lipids, fewer lipid droplets (LDs), and cell cycle arrest, indicating that PMP70 downregulation resulted in metabolic impairment. In conclusion, our study unveiled the pivotal role of PMP70-mediated peroxisomal functions in conferring chemoresistance, particularly in the context of LOHP resistance in CRC.

## Introduction

Metabolic reprogramming and deregulation are hallmark features of cancer. Alterations in lipid metabolism have gradually been proven to be important adaptations that support tumor progression, metastasis, and therapy resistance, rather than merely passive processes [1–3]. Several studies have validated that enhanced lipogenesis, lipid catabolism, and LD formation and turnover have emerged as key features of therapy-resistant tumor cells [4–8]. Targeting aberrant lipid metabolism could serve as a promising therapeutic strategy for overcoming therapy resistance.

Peroxisomes are highly metabolic organelles present in most eukaryotic cells, well-known for their roles in lipid metabolism [9]. The very long chain fatty acids that have 22 or more carbons undergo β-oxidation in peroxisomes before being oxidized in the mitochondria [9–11]. The β-oxidation process in peroxisomes generates reactive oxygen species (ROS), which is mainly eliminated by catalase (CAT). As highly dynamic organelles, peroxisomes can modify their shape, size, location, and abundance in response to nutritional or environmental signals. For example, under hypoxic conditions (1% oxygen), peroxisomal biogenesis genes and plasmalogen synthesis genes are selectively essential for cell survival [12]. Under nutrient stress, the physical interaction between peroxisomes and LDs increases significantly, leading to the release of fatty acids through lipolysis in white adipose tissue [13]. Peroxisomal plasticity thus becomes a powerful weapon for cells in resisting external stresses.

Peroxisomal dysfunction has also been implicated in tumor development. Notably, a reduction in peroxisome abundance has been reported in several neoplastic tissues, including colon carcinoma [14]. Recent studies have shown that genes involved in peroxisomal β-oxidation, such as NUDT7 and ACOX1, can suppress CRC progression [15, 16]. And our previous work has reported that CRC patients with more active peroxisome pathways had favorable overall survival [17]. All these suggest that peroxisome potentially may act as a tumor-suppressive organelle in CRC. In addition, recent studies have highlighted the role of peroxisomal activity in influencing the response to systemic therapies in breast cancer and lymphoma, indicating that peroxisomes adapt to therapeutic stress, thereby protecting cells from therapy-induced cell death [18–20]. Therefore, these findings underscore the need for further investigation into the distinct functions of peroxisomes in different contexts, particularly in CRC.

PMP70 is a 70 kDa protein locates on the peroxisomal membrane encoded by the ATP Binding Cassette Subfamily D Member 3 (*ABCD3*) gene. It is the most enriched peroxisomal membrane protein and the first observed peroxisomal ATP Binding Cassette(ABC) transporter. It participates in the peroxisomal import of dicarboxylic acids, branched-chain fatty acids, and C27 bile acid intermediates [21]. PMP70 is commonly recognized as a specific marker of peroxisomes. Clinical evidence showed that ABCD3 mRNA and protein levels are reduced in CRC tumor tissues, and low ABCD3 mRNA levels are correlated with poor prognosis in CRC patients [22], suggesting a potential tumor-suppressive role for PMP70 in CRC. However, *ABCD3* amplification has been observed in 19 drug-resistant cancer cell lines [23]. These findings highlight the potential dual role of PMP70, which may act as a tumor suppressor in CRC while also being involved in adaptive mechanisms under therapeutic pressure. This duality underscores the need to investigate its specific functions in CRC, particularly in the context of chemoresistance.

In this work, we found that peroxisome abundance and PMP70 level were associated with LOHP treatment recurrence in CRC patients. Knockdown of PMP70 significantly overcame LOHP resistance in CRC cells. Our findings unveil that peroxisomes and PMP70 play a significant role in conferring chemoresistance in CRC.

## Materials and methods

### Human CRC specimens and RNA-seq

Human colorectal cancer specimens were obtained from the Department of Colorectal and Anal Surgery at Zhongnan Hospital of Wuhan University. The tissue sections were stained with H&E and diagnosed through pathological examination. Our study was approved by the Ethics Committee of Zhongnan Hospital of Wuhan University (No. 2020106,2020-05). All patients signed written informed consent forms. Clinical characteristics of our CRC RNA-seq cohort are shown in Supplementary Table 1. RNA samples from clinical CRC tissues and cells (*n* = 3) were assessed for integrity using RNA Nano 6000 (Agilent Technologies, CA, USA) and then subjected to library construction. The libraries were sequenced on Illumina Novaseq 6000 using RNA sequencing technology provided by Novogene (Beijing, China).

### Cell culture

Human HCT116, HCT8, HT29, DLD1, SW620, SW480, and RKO cells were obtained from the American Type Culture Collection (ATCC) and we cultured them in ATCC-recommended medium supplemented with 10% fetal bovine serum (Gibco, NY, USA) and 1% penicillin-streptomycin (Gibco). The LOHP-resistant cell lines, HCT116L and HCT8L, were developed as previously described [24]. These cell lines were generated by exposing the cells to increasing concentrations of LOHP (ranging from 0.1 μmol/L to 2 μmol/L), which allowed for the selection of LOHP-resistant cells capable of being cultured in 2 μmol/L LOHP. LOHP (HY-17371), Nec1s (Necrostatin 2 racemate, HY-14622A), and NAC (Acetylcysteine, HY-B0215) were obtained from MedChemExpress (MCE). Lip-1 (Liproxstatin-1, S7699) and Z-VAD-fmk (S7023) were purchased from Selleck.

### Lipid ROS assay and cell cycle profiling

Cells were seeded on 12-well plates and incubated overnight. The following day, cells were treated with compounds for the specified duration, harvested by trypsinization, and suspended in 200 μL of PBS containing 5 μM C11-BODIPY 581/591 (Invitrogen). After a 30-min incubation at 37 °C in a water bath, cells were washed three times with PBS. LPO was evaluated using the Beckman CytoFLEX S Flow Cytometer equipped with a 488 nm laser on an FL1 detector. At least 10,000 single cells were analyzed per well. For cell cycle profiling, cells were seeded in 6-well plates to achieve 70–80% confluency at the time of harvest. Cells were fixed in 70% ethanol and stored at –20 °C for up to two weeks. The cell cycle was analyzed using FxCycle™ PI/RNase Staining Solution (Thermo, F10797) and quantified by Flow Cytometer.

### Mitochondrial ROS and stress profiling

MitoSox Red (Thermo Fisher Scientific, M36008) was used to measure mitochondrial superoxide levels. Cells were incubated with 5 µM MitoSox Red in PBS at 37 °C for 30 min in the dark. After washing with PBS, fluorescence was measured using a flow cytometer (Ex/Em: 510/580 nm). Mitochondrial function was assessed using the Seahorse XF Mito Stress Test Kit (Agilent). Cells were seeded in XF96 plates, equilibrated with XF Assay Medium, and treated sequentially with oligomycin (1 µM), FCCP (2 µM), and rotenone/antimycin A (0.5 µM). Oxygen consumption rate (OCR) was measured, and parameters such as basal and maximal respiration were calculated using Seahorse Wave software.

### Untargeted lipidomic analysis

Lipids were extracted from the organic phase using a modified Bligh and Dyer method [25]. The lipids were then separated and analyzed using a Qexactive mass spectrometer (Thermo Fisher Scientific, USA) with a CSH C18 column (1.7 mm × 2.1 × 100 mm, Waters, USA). Elution was performed using the following gradient: 0–2 min at 40–43% liquid B (10 mM ammonia formate, 0.1% formic acid, 90% isopropyl alcohol, and 10% acetonitrile); 2–2.1 min at 43–50% liquid B; 2.1–7 min at 50–54% liquid B; followed by an increase to 70%-99% from 7 .1 to 13 min. Flow rate was set to 0.35 mL/min. For positive/negative ionization modes, the spray voltage was set to 3.8/-3.2 kV respectively while maintaining the aux gas heater temperature at 350 °C and capillary temperature constant at 320 °C. The full scan range of m/z values ranged from 200 to 2000 with a resolution of 70,000 and AGC target for MS acquisitions set to 3,000,000 with maximum ion injection time of 100 ms. For subsequent MS fragmentation, the top three precursors were selected with a maximum ion injection time of 50 ms, resolution of 17,500, and AGC fixed at 100,000. Stepped normalized collision energy was set to 15, 30, and 45 eV. Lipid identification and quantitation were performed using LipidSearch 4.1 SP2 (Thermo Fisher Scientific Inc., USA), followed by data scaling and normalization using metaX.

### Mouse experiments

We purchased male athymic nude mice (3 weeks old) from GemPharmatech (Jiangsu, China). The Zhongnan Hospital of Wuhan University Institutional Animal Care Animal Welfare Committee approved the experiments. We implanted sgNC and sgPMP70#2 HCT116L cells (3 × 10^6^ cells/0.1 ml) subcutaneously in the right side of the dorsal midline of the mice (8 weeks old). Once tumor volume reached 80–180 mm^3^, we randomly assigned mice to different treatment groups and treated them with vehicle or LOHP (5 mg/kg, i.p., twice a week) for 20 days. After drug administration, we measured tumors every three days and sacrificed the mice at day 42 (after injection of cells).

### Statistical analysis

Peroxisome pathway score was calculated as previously described [17]. To determine the PMP70 protein score, we categorized CRC patients into two groups based on WB results: PMP70-High and PMP70-Low. Random Forest (RF) was used, a commonly used ensemble machine learning algorithm [26], to establish a prediction model using RNA-seq data from these patients. GraphPad Prism 8 was used to derive *P* values, which are indicated as *P < 0.05, **P < 0.01 and ***P < 0.001. We employed *t* test, one or two-way ANOVA, and Dunnett’s or Bonferroni’s post hoc test to obtain these values.

### Additional materials and methods

Further details on the methods can be found in the Supplementary Methods. The antibodies and siRNAs are provided in Supplementary Tables S2–S3. Original data for western blot analyses are included as original data 1.

## Results

### Peroxisome abundance correlates with LOHP-based chemotherapy in CRC

**Fig. 1:**
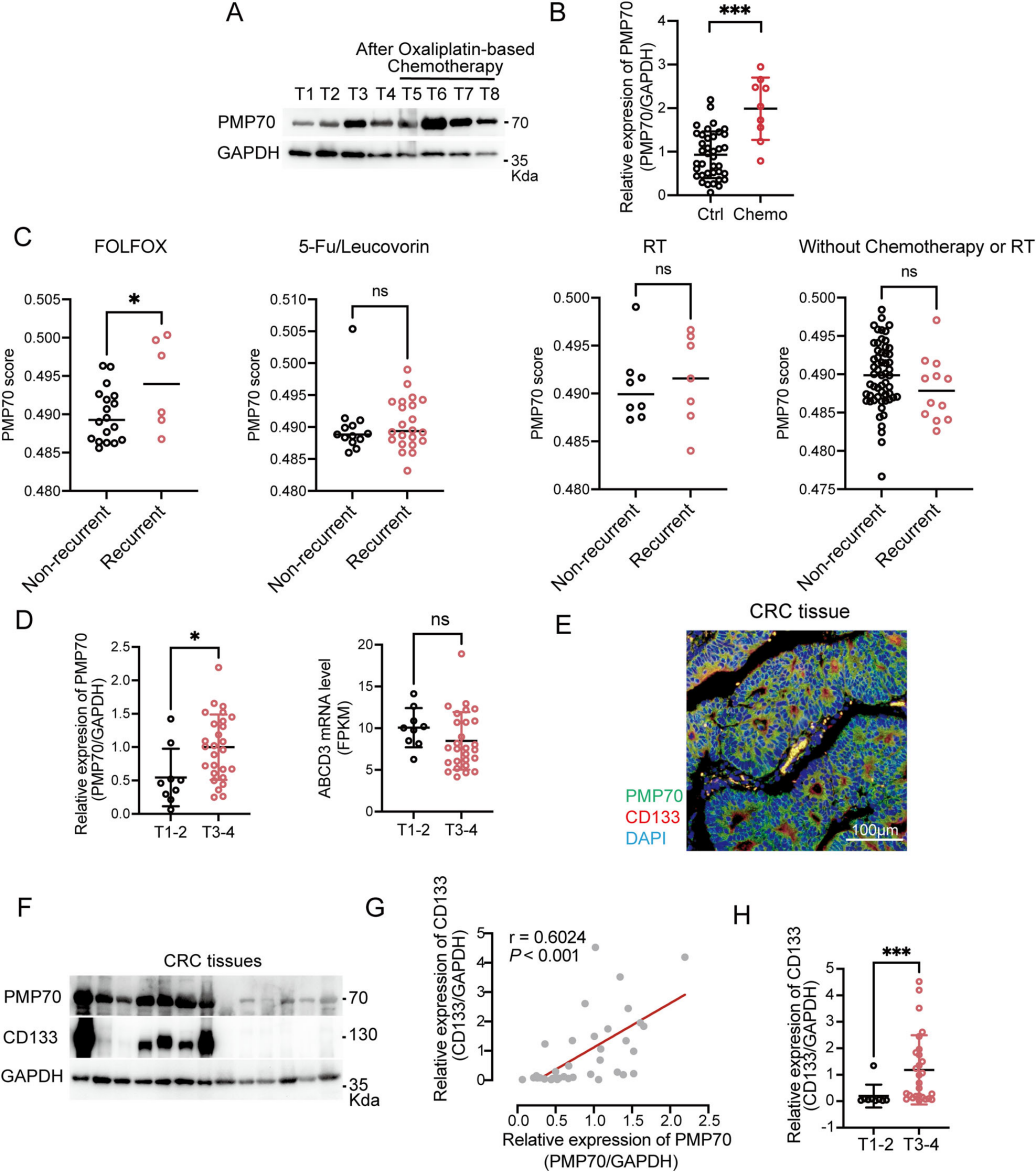
Peroxisome abundance and PMP70 protein levels correlate with CRC progression and LOHP treatment. **A**, **B** Western blot analysis and quantification of PMP70 protein levels in 38 primary tumor tissues and 9 LOHP-treated CRC tumor tissues. Protein levels were quantified using ImageJ. **C** PMP70 scores in CRC primary tumor tissues with and without recurrence, subgrouped by treatment types in the GSE106584 dataset. FOLFOX: LOHP + 5FU+Leucovorin; RT: Radiotherapy. **D** Western blot analysis of PMP70 protein levels and RNA-seq analysis of ABCD3 mRNA expression in 38 CRC primary tumor tissues at different T stages. Protein levels were quantified with ImageJ. **E** Representative immunofluorescence images showing colocalization of PMP70 and CD133. **F** Western blot analysis of PMP70 and CD133 protein levels in 13 CRC primary tumor tissues. **G** Correlation analysis of PMP70 and CD133 protein levels in 38 CRC tumor tissues based on ImageJ quantification of Western blots. **H** CD133 protein levels in CRC tumor tissues at early versus advanced T stages. *P < 0.05, **P < 0.01, ***P < 0.001.

To explore the potential role of peroxisome in CRC, we measured the expression of peroxisomal marker PMP70 in CRC tumor tissues and investigated the correlation between clinical characteristics and PMP70 level. Western blotting analysis revealed elevated levels of PMP70 in CRC tumors went through LOHP-based chemotherapy compared to the untreated tumors (Figs. 1A, B). To further investigate the correlation between peroxisomes and LOHP therapy in CRC, a systemic treatment database (GEO Number: GSE106584) was incorporated into our study. Since neither ABCD3 mRNA nor genes in the peroxisome pathway reliably reflect peroxisome abundance, the random forest algorithm was employed to generate a PMP70 score, which could be used to predict the likelihood of peroxisome abundance in the tissues. The prediction was based on data from our CRC cohort, which included RNA-seq data along with the matched PMP70 protein levels (Fig. S1A). Analyzing the dataset by this method, we found that within the FOLFOX treatment group (LOHP, 5-Fu, and Leucovorin), the PMP70 scores of the tissues from the recurrent CRC subgroup were remarkably higher. This trend was not evident in the 5-Fu/Leucovorin, radiotherapy, or untreated groups. These findings strongly implied that higher peroxisome abundance was associated with the resistance to LOHP treatment in CRC (Fig. 1C).

Intriguingly, there is a significant increase in PMP70 protein levels in tumor tissues of CRC patients with higher T stages, while the mRNA levels of ABCD3 do not show a similar elevation (Fig. 1D). Cancer stem cells (CSCs) were involved in tumor initiation, progression, recurrence and therapy resistance [27]. A previous work has reported that peroxisome abundance was elevated during colon stem cell differentiation [28]. Therefore, expression of CSCs marker CD133 was examined in CRC tumor tissues. Immunofluorescence staining showed CD133 was expressed and partially colocalized with PMP70 in the CRC tumor (Fig. 1E). The protein levels of PMP70 were positively correlated with CD133 (Fig. 1F, G), both of which were upregulated in CRC tissues at advanced T stages (Fig. 1H).

### LOHP-resistant CRC cells are resistant to pexophagy

**Fig. 2:**
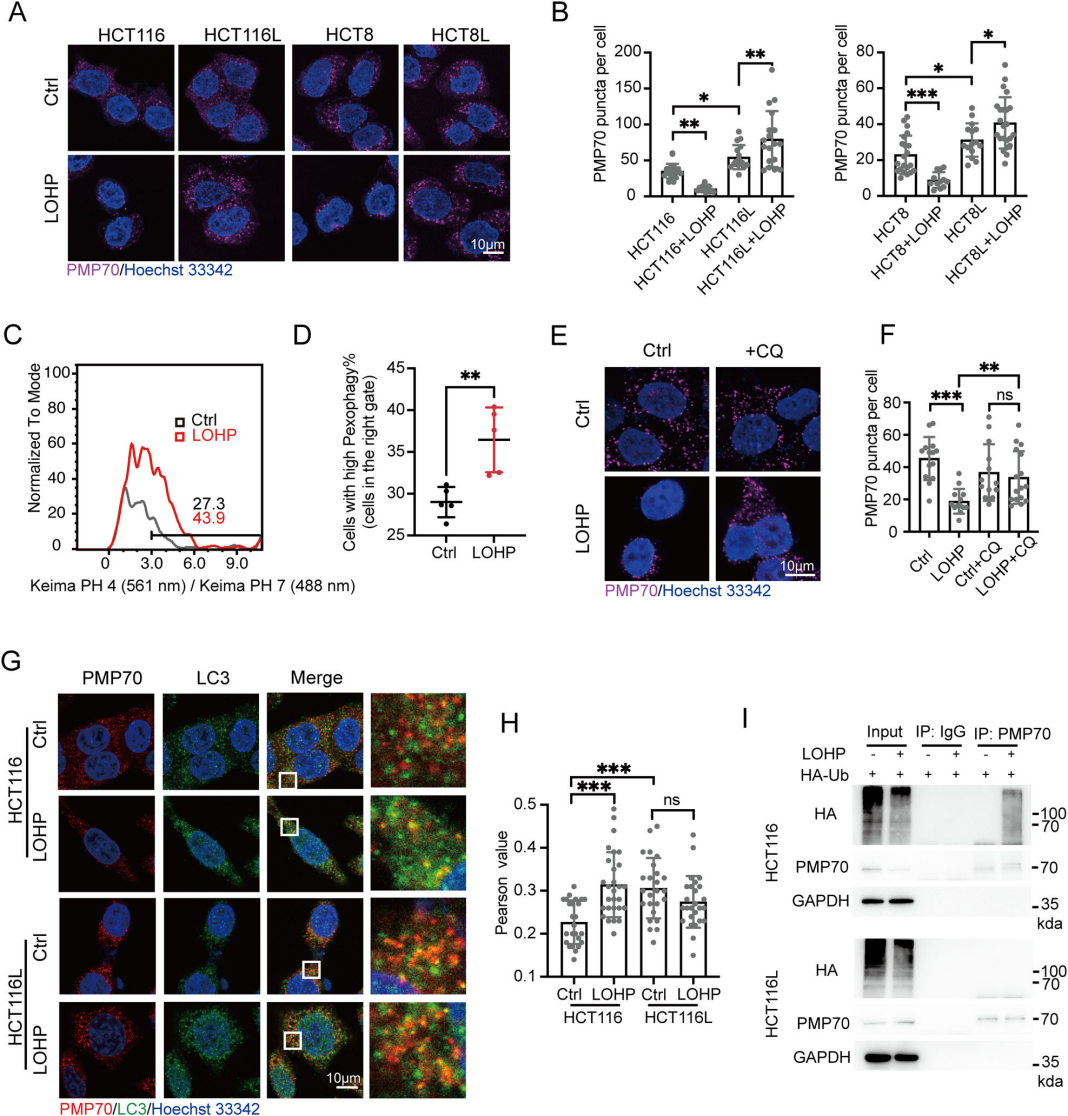
LOHP-resistant CRC cells exhibit reduced LOHP-induced pexophagy. **A** Representative confocal images of PMP70 puncta in parental and LOHP-tolerant CRC cells treated with LOHP. **B** Quantification of PMP70 puncta in panel (**A**). **C** Confocal images of HCT116 cells expressing dKeima-SKL with or without LOHP treatment (60 μM). **D** Flow cytometry analysis and quantification of HCT116 cells expressing dKeima-SKL treated with LOHP (40 μM). Data represent 4–5 images and >15 cells per condition. **E**, **F** Confocal images and quantification of PMP70 puncta in HCT116 cells treated with vehicle or 10 μM CQ in the presence or absence of LOHP. Data represent 5–6 images and >25 cells per condition. **G**, **H** Colocalization of PMP70 puncta and LC3 puncta in HCT116 and HCT116L cells, both treated by vehicle and 60 μM LOHP. Panel (**H**) is the quantification of panel G. 5 ~ 6 images, total of 25+ cells counted per condition. **I** Ubiquitinated-PMP70 increased upon LOHP treatment in HCT116 than in HCT116L cells. HCT116 and HCT116L cells were transfected with HA-Ub for 24 h and then treated with LOHP (60 μM) for 24 h. Co-immunoprecipitation of PMP70 with HA, and corresponding inputs. *P < 0.05, **P < 0.01, ***P < 0.001.

For generating an in vitro model of LOHP-resistant CRC, we treated HCT116 and HCT8 CRC cell lines with low-dose LOHP for several rounds. The selected cells termed as HCT116L and HCT8L, which showed high resistance to LOHP treatment (Fig. S1B). Peroxisome-resident proteins, including PEX3, PEX16, CAT, and PMP70, were all upregulated in HCT116L comparing to HCT116 (Fig. S1C). The increase of peroxisome, indicated by PMP70, was also proved through imaging as HCT116L and HCT8L had significantly higher levels of PMP70 puncta than parental cells. Driving for peroxisomal changes in responding to LOHP treatment in LOHP-resistant and parental CRC cells, we further treated the cells with LOHP. Unexpectedly, we observed a significant decrease of PMP70 puncta in HCT116 and HCT8 but an opposite increase of it in LOHP-resistant cells upon LOHP treatment (Fig. 2A, B), suggesting peroxisomal abundance increase might be critical to the CRC cells in responding to the LOHP-caused stress.

Peroxisome homeostasis is mainly regulated by pexophagy, a type of selective autophagy process that degrades peroxisomes [29]. To characterize whether the peroxisome abundance decrease in HCT116 caused by LOHP treatment is mediated by pexophagy or not, we generated the Keima-SKL expression HCT116 cell line, in which the SKL sequence leads a pH-sensitive reporter Keima locating on the peroxisome. LOHP treatment increased the Ex.561/Ex.488 (acidic environment/neutral environment) ratio of Keima-SKL protein, suggesting that LOHP treatment induced pexophagy in HCT116 (Fig. 2C, D). Besides, the loss of peroxisomes caused by LOHP treatment was completely rescued by chloroquine (CQ), an autophagy inhibitor blocking autophagosome-lysosome fusion (Fig. 2E, F). These data uncovered that LOHP induced pexophagy and the subsequent loss of peroxisomes in HCT116.

Since the peroxisome abundance of HCT116L would not decrease upon LOHP treatment, we hypothesized that HCT116L is resistant to LOHP-induced pexophagy. LOHP treatment significantly increased the colocalization of PMP70 puncta and LC3 puncta in HCT116, but not in HCT116L (Fig. 2G, H). The ubiquitination of peroxisomal proteins, such as PMP70, which can be recognized by autophagy receptors, is commonly served as a signal for pexophagy. We found that PMP70 is ubiquitinated upon LOHP treatment in HCT116, but not in HCT116L (Fig. 2I). Together, these results demonstrated that HCT116L was resistant to LOHP-induced pexophagy.

### PMP70 depletion overcome LOHP resistance in CRC cells

**Fig. 3:**
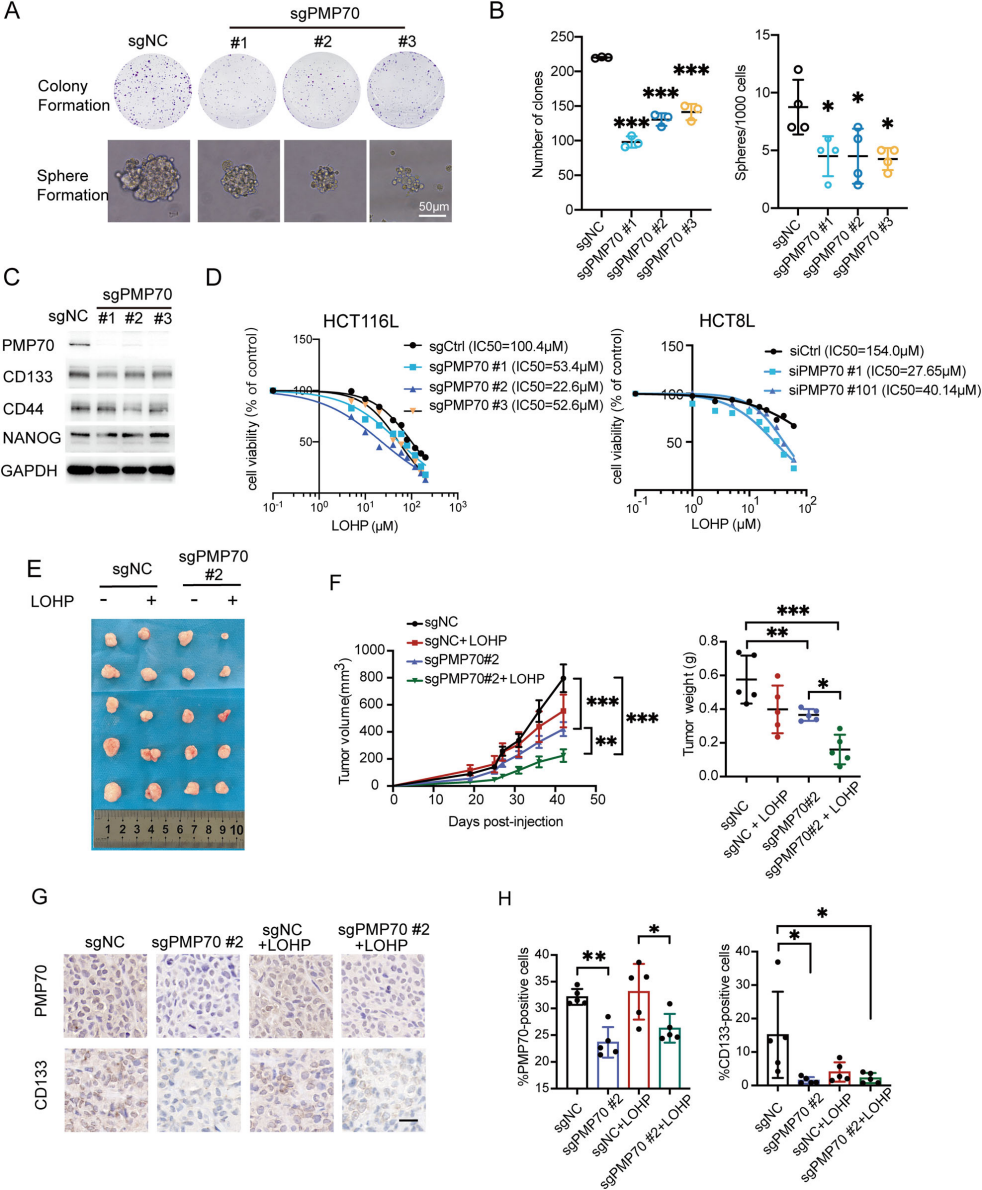
Silencing PMP70 overcomes LOHP resistance. Representative images (**A**) and quantification (**B**) of colony formation and sphere formation assays of PMP70-depleted and control HCT116L cells. **C** Western blot analysis of PMP70, CD133, CD44, and Nanog expression in sgPMP70 and sgNC HCT116L cells. **D** Effects of LOHP on cell survival measured by CCK8 assays in sgNC and sgPMP70 HCT116L cells (left panel). Survival curves for HCT8L cells transfected with siRNA targeting PMP70 or control siRNA in response to LOHP (right panel). The IC50 of LOHP for each condition is indicated in the plots. **E** Representative images of xenograft tumors derived from sgPMP70 or sgNC HCT116L cells treated with LOHP or vehicle. **F** Tumor growth curves and tumor weight quantification in different treatment groups. **G** IHC staining of PMP70 and CD133 in tumor tissues from control or LOHP-treated mice bearing sgNC or sgPMP70 HCT116L xenografts. **H** Quantification of panel (**G**). *P < 0.05, **P< 0.01, ***P < 0.001.

To investigate PMP70 functions in LOHP-resistant CRC cells, PMP70-depleted HCT116L cell lines were generated using CRISPR-Case9 interference. PMP70 depletion significantly reduced the proliferation and sphere formation in HCT116L cells (Fig. 3A, B). PMP70-depletion also led to a decrease of CD133 at the protein level (Fig. 3C). Notably, knocking out PMP70 enhanced the efficacy of LOHP in HCT116L and HCT8L cells (Fig. 3D).

We further tested the response of PMP70-depleted HCT116L cells to LOHP in vivo. HCT116L transfected with non-targeting sgRNA (sgNC) and sgPMP70 were subcutaneously injected in the right flank of nude mice, respectively. PMP70-depleted tumors showed minor regression compared to control tumors, and PMP70 depletion enhanced the tumor-suppression efficacy of LOHP (Fig. 3E, F). Immunohistochemical (IHC) staining confirmed that PMP70-depleted tumors exhibited a significantly lower number of PMP70-positive cells compared to control tumors. A decreased level of CD133 positive cells was also observed in PMP70-depleted tumors (Fig. 3G, H), which was consistent with the findings in vitro. These data suggest that PMP70 depletion overcame LOHP resistance in HCT116L, both in vitro and in vivo.

### PMP70 protect CRC cells from LPO

**Fig. 4:**
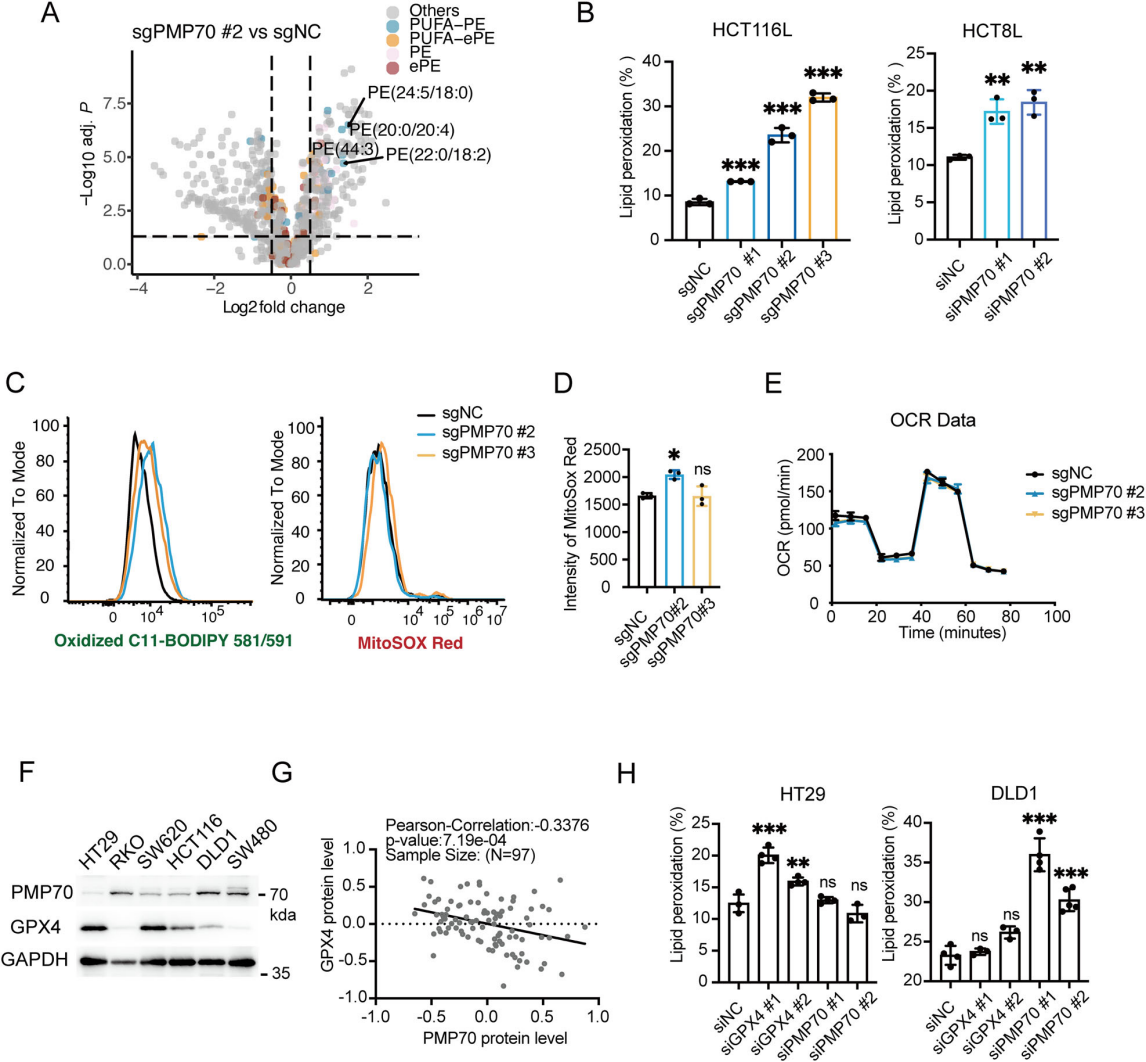
PMP70 protects CRC cells from LPO. **A** Volcano plot showing lipid species significantly altered between sgPMP70#2 and control HCT116L cells. PE: phosphatidylethanolamine; ePE: ether-PE; PUFA: polyunsaturated fatty acid. **B** Lipid ROS levels measured using BODIPY 581/591-C11 in sgPMP70 and control HCT116L cells, as well as in HCT8L cells transfected with siRNA targeting PMP70 or control siRNA. **C** Fluorescence histograms of BODIPY 581/591-C11 and MitoSOX Red staining in sgPMP70 and control HCT116L cells, illustrating LPO and mitochondrial ROS levels, respectively. **D** Quantification of mean fluorescence intensity of MitoSOX Red staining in sgPMP70 and control HCT116L cells. **E** Mitochondrial stress profile of sgPMP70 and control HCT116L cells, assessed using Seahorse extracellular flux analysis. **F** Western blot analysis of PMP70 and GPX4 in various CRC cell lines. **G** Correlation between PMP70 and GPX4 protein levels in the CPTAC-COAD dataset (*n* = 97). **H** LPO in HT29 and DLD1 cells transfected with siRNA targeting PMP70 (siPMP70#1, #2), GPX4 (siGPX4#1, #2), or control siRNA. *P < 0.05, **P < 0.01, ***P < 0.001.

Since PMP70 is a peroxisomal membrane protein essential for lipid transportation and metabolism, we compared the lipidome of PMP70-depleted and control HCT116L cells, and found that the abundance of several polyunsaturated phosphatidylethanolamines (PUFA-PE) was significantly higher in PMP70-depleted HCT116L cells (Fig. 4A). Since PUFA-PE was known as the main substrate of LPO, we hypothesized that PMP70 depletion would change the LPO level in cells and used BODIPY-C11 probe to verify it. PMP70-depleted HCT8L and HCT116L cells exhibited higher LPO levels compared to their parental counterparts (Fig. 4B). Metabolic interplay between peroxisomes and mitochondria is crucial for cellular metabolic homeostasis [9, 30–32]. Therefore, we further tested mitochondrial functions. Results showed that mitochondrial ROS was comparable between PMP70-depleted cells and control cells (Fig. 4C, D), and mitochondria functional assay showed no obvious change in PMP70-depletion cells (Fig. 4E). These results suggest that PMP70 deletion induced excessive LPO without influencing mitochondrial stress in LOHP-resistant CRC cells.

GPX4 is a critical enzyme for the primary reduction of lipid peroxides. The protein levels of PMP70 and GPX4 were examined in several CRC cell lines. Of note, CRC cells with higher expression of PMP70 (PMP70^*High*^; DLD1, SW480, RKO) exhibited relatively lower level of GPX4 and vice versa (Fig. 4F). Proteomic data of 97 colon tumor tissues from TCGA-CPTAC dataset validated the negative correlation of PMP70 and GPX4 protein level (Fig. 4G, r = –0.3376, *P* = 0.0007). We then hypothesized that PMP70 and GPX4 might be complementary factors in CRC cells for preventing LPO. PMP70 and GPX4 were knockdown in HT29 and DLD1 cells, respectively. GPX4 knockdown induced LPO in HT29 cells (PMP70^*Low*^) but not in DLD1 cells (PMP70^*High*^); PMP70 knockdown increased LPO in DLD1 but not in HT29 (Fig. 4H). Treatment with the GPX4 covalent inhibitor 1S,3R-RSL3 (RSL3) in control and PMP70-depleted (or -knockdown) PMP70^*High*^ cells (DLD1 and HCT116L) did not significantly increase the sensitivity of the cells to RSL3-induced LPO and cell death (Fig. S2). Together, these results suggest that PMP70 might play an important role in preventing LPO in PMP70^*High*^ CRC cells.

### Silencing PMP70 enhances LOHP-induced LPO in CRC cells

**Fig. 5:**
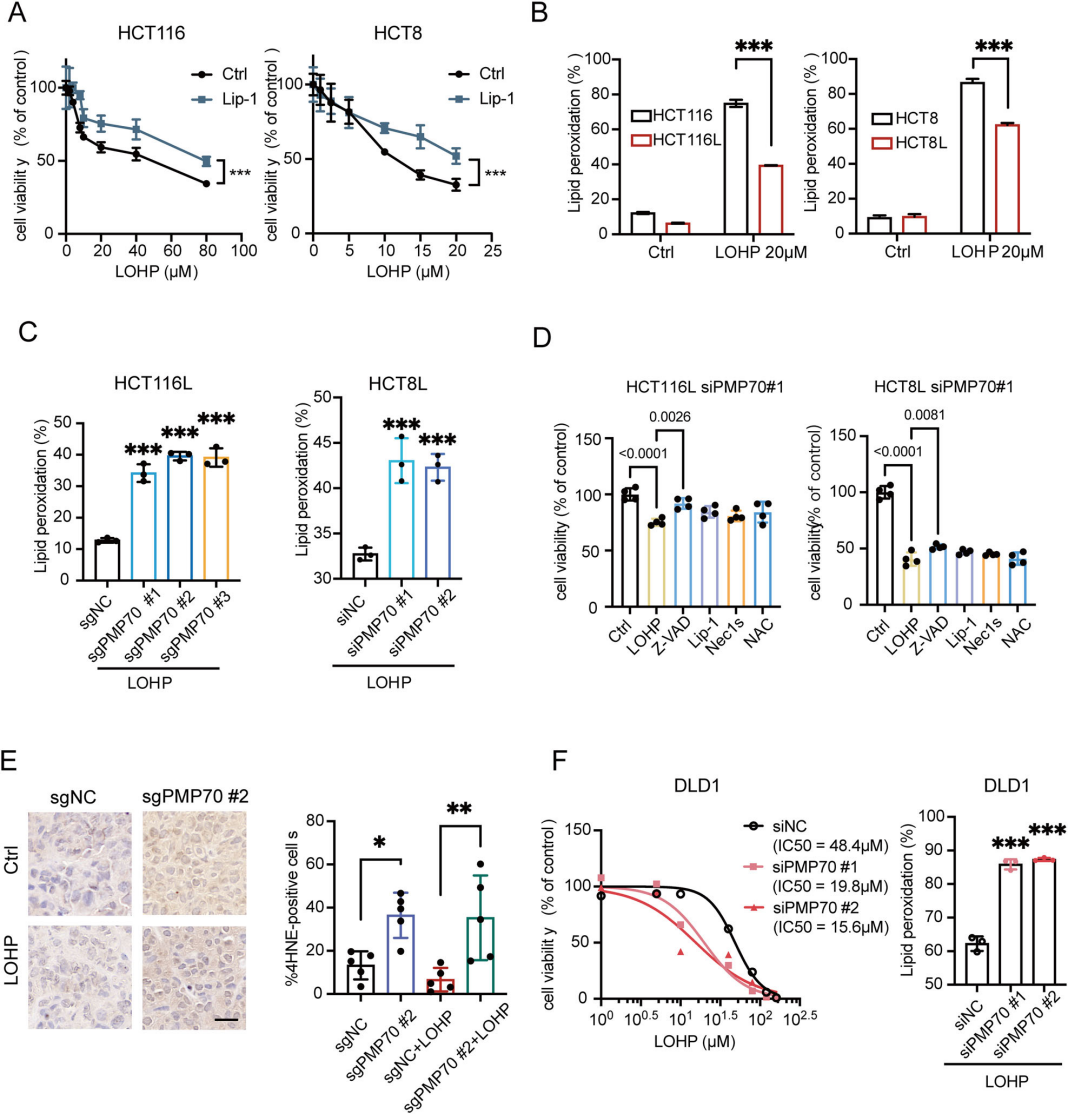
Silencing PMP70 enhances LOHP-induced LPO in CRC cells. **A** Cell viability of HCT116 and HCT8 cells treated with liproxstatin-1 (Lip-1, 2 μM) under different doses of LOHP (*n* = 6). **B** Percentage of oxidized lipids measured by BODIPY 581/591-C11 in HCT116L, HCT8L, and their parental cells, with or without LOHP treatment (20 μM, 48 h). **C** LPO levels in HCT116L cells transduced with sgPMP70 or sgNC and in HCT8L cells transfected with siPMP70 or siNC after LOHP treatment (10 μM). **D** Cell viability of siPMP70#1 and control cells treated with LOHP (HCT8L:20 μM, HCT116L:40 μM) and LOHP combined with different cell death inhibitors (2 μM Lip-1, 10 μM Z-VAD-fmk, 1 μM Nec1s, and 10 μM NAC). **E** 4-HNE IHC staining of control or LOHP-treated mice bearing HCT116L tumors transduced with sgNC or sgPMP70 (*n* = 5). Right chart: quantification of the staining intensity using ImageJ. **F** Survival curve and LPO levels in siPMP70 or control DLD1 cells after LOHP treatment (20 μM). *P < 0.05, **P < 0.01, ***P < 0.001.

Since peroxisome protein PMP70 can reduce LPO level, and LOHP treatment would reduce peroxisome abundance (Figs. 4B, 2B), we investigated whether LOHP treatment induces LPO in CRC. Liproxstatin-1 (Lip-1) was used to trap lipophilic radicals in HCT116 and HCT8 cells. As shown, Lip-1 partially rescued LOHP-induced cell death (Fig. 5A), suggesting that LPO may be one of the mechanisms underlying LOHP-induced cell death. Flow cytometry analysis also revealed that the LOHP treatment-induced increase in LPO levels was significantly lower in LOHP-resistant cells compared to LOHP-sensitive cells (Fig. 5B).

We then investigated the responses of PMP70-interfered LOHP-resistant cells to LOHP-induced LPO and cell death, both in vitro and in vivo. Knockdown of PMP70 significantly enhanced LOHP-induced LPO in HCT116L and HCT8L cells (Fig. 5C). Furthermore, we tried to investigate the type of cell death induced by PMP70 knockdown under LOHP treatment in HCT116L and HCT8L. However, the sensitivity of resistant cells to LOHP-induced cell death cannot be fully rescued by any of the cell death inhibitors tested, even though apoptosis inhibitors can slightly restore cell viability. This phenomenon suggests that the increased sensitivity to drugs induced by PMP70 knockdown do not follow a known form of programmed cell death, indicating a more complex underlying mechanism (Fig. 5D). In xenograft tumors, LPO was assessed by IHC staining of 4-HNE, a major end-stage product of LPO. PMP70-depleted tumors exhibited a higher number of 4-HNE-positive cells compared to control tumors (Fig. 5E), further supporting the role of PMP70 in modulating LPO. Additionally, downregulation of PMP70 in PMP70^*High*^ CRCs (DLD1) significantly enhanced the cell-killing efficacy of LOHP and increased LOHP-induced LPO (Fig. 5F). Together, all these results suggest that silencing PMP70 could enhance LOHP treatment-caused LPO and its killing efficacy in LOHP-resistant and PMP70^*High*^ CRC cells.

### PMP70 stabilizes neutral lipids and LDs in LOHP-resistant CRC cells

**Fig. 6:**
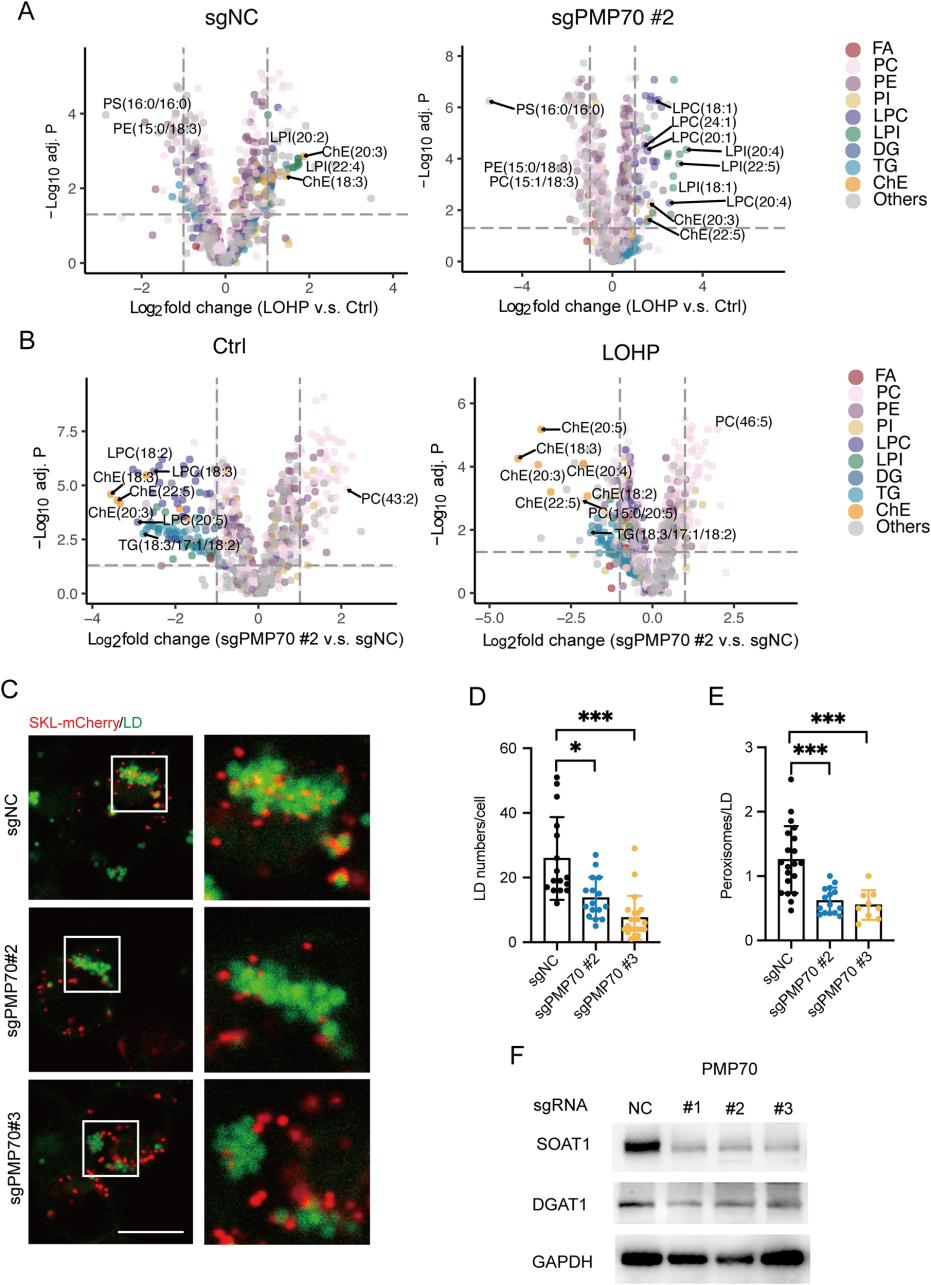
PMP70 stabilizes neutral lipids and LDs in LOHP-resistant CRC cells. **A** Volcano plots of lipid species significantly altered by LOHP compared to vehicle treatment in sgPMP70 #2 (right panel) and sgNC (left panel) HCT116L cells. LPI: lysophosphatidylinositols; ChE: cholesteryl ester; LPC: lysophosphatidylcholine; PS: phosphatidylserine; PC: phosphatidylcholine; PE: phosphatidylethanolamine. **B** Volcano plots of lipid species significantly altered between sgPMP70 #2 and sgNC HCT116L cells under LOHP (right panel) or vehicle treatment (left panel). TG: triacylglycerol. **C**–**E** HCT116L cells transduced with sgPMP70 (#2, #3) or sgNC were transfected with mCherry-SKL for 48 h. Scale bar = 10 μm. LDs were stained with Lipi-Blue (100 nM, Dojindo) for 30 min and analyzed by confocal microscopy (panel **C**). Quantification of LDs per cell is shown in panel (**D**). Quantification of peroxisome-LD contacts per cell is shown in panel (**E**). **F** Immunoblots of SOAT1, DGAT1, and GAPDH in sgPMP70 and sgNC HCT116L cells. *P < 0.05, **P < 0.01, ***P < 0.001.

We further conducted a lipidomic analysis to investigate the altered lipidome of PMP70-depleted and control cells upon LOHP treatment. LOHP treatment upregulated 4 out of 6 cholesteryl esters (ChEs) and part of lysophosphatidylinositols (LPIs) in HCT116L cells (Fig. 6A). In PMP70-depleted HCT116L cells, most lysophosphatidylcholines (LPCs) and LPIs increased, and only 2 out of 6 ChEs significantly increased upon LOHP treatment. Compared to the control HCT116L cells, most neutral lipids, including ChEs and triacylglycerols (TGs), were significantly decreased in PMP70-depleted HCT116L cells with or without LOHP treatment (Fig. 6B).

Neutral lipids are mainly stored in LDs in the cells. Therefore, we used Lipi-blue staining to evaluate LD abundance of PMP70-depleted and control HCT116L cells. Consistent with the lipidomic results, PMP70 depletion caused significant reduction of LDs in LOHP-resistant cells (Fig. 6C, D). Critical enzymes for neutral lipid synthesis and LD formation, including SOAT1 and DGAT1, were reduced in PMP70-depleted HCT116L cells (Fig. 6F). Unexpectedly, a decrease in peroxisome-LD connections was also observed in PMP70-depleted cells (Fig. 6C, E). These data indicate that PMP70 contributes to the LD homeostasis regulated by peroxisomes in LOHP-resistant CRC cells.

### PMP70 deletion leads to cell cycle arrest

**Fig. 7:**
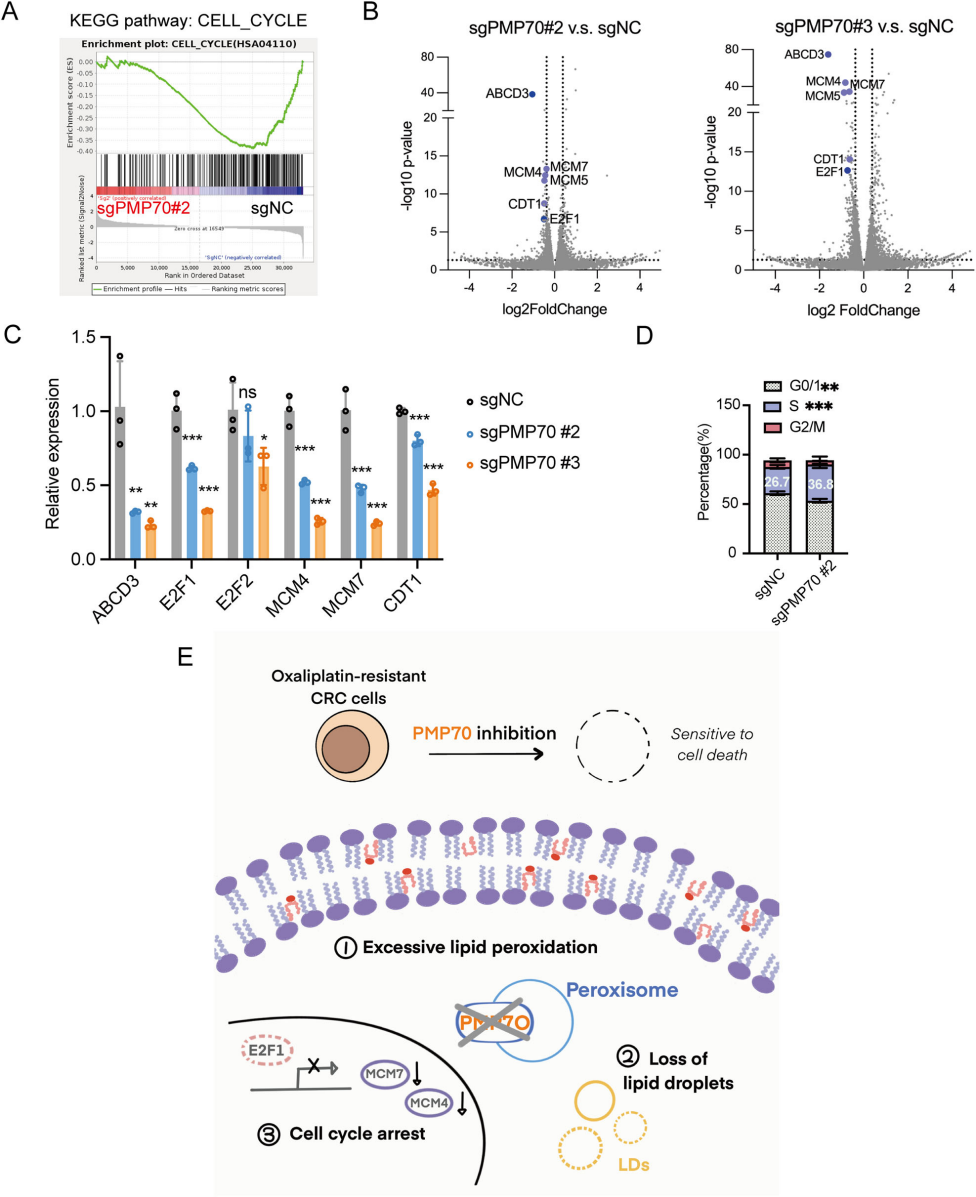
PMP70 deletion led to cell cycle arrest in LOHP-resistance CRC cells. **A** Gene Set Enrichment Analysis (GSEA) showing the downregulated cell cycle pathway (KEGG HSA04110) in sgPMP70#2 vs. sgNC cells. **B** Volcano plots showing significantly differentially expressed genes between sgPMP70#2 or sgPMP70#3 and sgNC cells. *ABCD3* and cell cycle-related genes (e.g., *E2F1*, *CDT1*, and *MCM* family members) are labeled. **C** Quantitative real-time PCR analysis of cell cycle-related gene expression in sgPMP70 or sgNC HCT116L. Data represent relative mRNA expression levels normalized to control. **D** Cell cycle distribution of sgPMP70#2 cells compared to sgNC, assessed by flow cytometry. The percentage of cells in G0/G1, S, and G2/M phases is shown. **E** Schematic representation of the effects and potential mechanisms underlying PMP70 downregulation in LOHP-resistant CRC cells. **p < 0.01, ***p < 0.001. Data are presented as mean ± SD.

To investigate the potential pathways impacted by PMP70 in LOHP-resistant CRC cells, we performed RNA-seq analyses on PMP70-depleted and control HCT116L cells. Pathway enrichment analysis revealed that the cell cycle pathway was significantly downregulated in PMP70-depleted cells (Fig. 7A). Among the most differentially expressed genes, apart from *ABCD3*, key regulators of DNA replication including *MCM4*, *MCM5*, *MCM7*, and *CDT1* were downregulated in PMP70-depleted cells (Fig. 7B). Of note, E2F1, a crucial transcription factor for cell cycle progression and proliferation, was also down-regulated due to PMP70 depletion. Changes of the expression levels of MCM4, MCM7, CDT1, and E2F1 were validated by qPCR (Fig. 7C). Consistently, flow cytometry analysis demonstrated that PMP70-depleted cells exhibited a prolonged S phase compared to the control cells (Fig. 7D). Together, these data suggest that PMP70 depletion in LOHP-resistant CRCs would cause the cell cycle arrest.

## Discussion

Recent studies have highlighted the role of peroxisomes in cancer biology, particularly in lipid metabolism and oxidative stress regulation. Peroxisomal regulators involved in β-oxidation, α-oxidation, and ether lipid synthesis have been shown to promote tumor progression in breast cancer, hepatocellular carcinoma, and prostate cancer [16, 33–35]. Catalase, a key peroxisomal ROS controller, is implicated in various cancers, as cancer cells often reduce ROS levels to avoid apoptosis [19, 36, 37]. Peroxisomes have also been linked to drug resistance. In lymphoma, inhibiting peroxisomal genes involved in biogenesis or pexophagy has been shown to enhance apoptosis in drug-resistant cells [19, 20]. These findings suggest that targeting peroxisomal function could be a potential strategy to overcome drug resistance and enhance therapy-induced cell death.

In CRC, peroxisomes are found to be reduced in tumor tissues, and certain proteins involved in their biosynthesis and β-oxidation act as tumor suppressors [38], suggesting a role in anti-tumorigenesis. Peroxisomes are versatile organelles with context-dependent functions, adapting to various cellular stresses. In the context of chemoresistance, our data suggested that peroxisomes may promote the survival of resistant CRC cells by shielding them from drug-induced killing. Specifically, we demonstrated that peroxisomes protect CRC cells from LOHP treatment, and silencing PMP70 can restore their sensitivity to LOHP.

We have explored peroxisome homeostasis in CRC chemoresistance. As data shown, LOHP triggered pexophagy resulting in the degradation of peroxisomes, whereas LOHP-resistant CRC cells show resistance to pexophagy induced by LOHP. Dahabieh et al. have revealed the role of pexophagy in therapy resistance [20]. They found that histone deacetylase inhibitor (HDACi) upregulated peroxisome abundance in lymphoma U937 cells, different from our findings in HCT116 cells. HDACi-resistant U937 cells possessed enriched peroxisomes as well as activated pexophagy, which is similar to the pattern observed in HCT116L cells, where we found resistant cells maintained stable and enriched peroxisomes and had higher colocalization of PMP70 puncta and LC3 puncta. They also reported that disrupting peroxisomal matrix protein transport through PEX26 silencing can induce pexophagy and contribute to delayed therapy resistance in lymphoma. The results we presented and previous reports suggest that drug-resistant cancer cells tightly regulate peroxisome homeostasis through enhanced pexophagy, despite differing reactions of peroxisomes in lymphoma and CRC cells when attacked by drugs. Since LOHP-resistant CRC cells tend to protecting and maintaining their abundant peroxisomes under LOHP treatment, disrupting peroxisome homeostasis may represent a promising strategy to eliminate these resilient cells.

Intriguingly, several PUFA-PEs, which are substrates of LPO, significantly increased in PMP70-depleted HCT116L cells that had elevated levels of LPO. Previously, Tabassum et al reported that LOHP can induce excess LPO [39]. Our study found that LPO is one of the mechanisms underlying LOHP-induced cell death in CRC. Silencing PMP70 in LOHP-resistant cells can induce overwhelming LPO in response to LOHP. This discovery suggested a new function for PMP70—protecting cells from LPO. GPX4 is a pivotal enzyme for preventing excess LPO in cells. Our data suggested that PMP70 plays a crucial role in limiting LPO in CRC cells with low GPX4 levels, where PMP70 is dominant. Besides GPX4, two other enzymes that play a crucial role in defending against LPO are ferroptosis suppressor protein 1 (FSP1) and dihydroorotate dehydrogenase (DHODH). Mao et al identified DHODH operates in parallel to mitochondrial GPX4 to inhibit LPO by reducing ubiquinone to ubiquinol and repressing DHODH induces extensive mitochondrial LPO in Low-GPX4 cancer cells [40]. Although our data suggests that PMP70 works in parallel with GPX4, further research are needed to understand the specific mechanisms by which PMP70 or peroxisomes defend against LPO.

To gain insight into the mechanism of PMP70 in preventing LPO, we conducted untargeted lipidomic analysis and RNA-seq analyses in PMP70-depleted HCT116L and control cells.

Firstly, we found that when PMP70 was silenced in HCT116L, numbers of LDs and most of the neutral lipids significantly decreased. Lippincott-Schwartz and coworkers validated that the transferring of fatty acids from LDs to peroxisomes requiring M1 Spastin tethering complex found in LDs and ABCD1 in peroxisomes [41]. The LD-peroxisome trafficking mediated by M1 Spastin and ABCD1 can relieve LPO in LDs. ABC transporters of subfamily D include four proteins: ABCD1, ABCD2, ABCD3 and ABCD4. Except for ABCD4, the other three are located on peroxisome membrane responsible for lipid transport. Our data also showed that depletion of PMP70 (ABCD3) in HCT116L cells caused a significant decrease of contacts between LDs and peroxisomes. These results indicates that peroxisomal lipid transporters, such as ABCD1 and ABCD3, may be important for communication between peroxisomes and LDs. Moreover, we identified that knockout of PMP70 decreased LD numbers and enzymes critical for LD biosynthesis in LOHP-resistant cells. In cancer cells, inhibition of LD synthesis can channel PUFA away from TAG into phospholipids and finally sensitize the cells to LPO [42, 43]. This may be a possible explanation of the enhanced sensitivity of sgPMP70 cells to LPO, since PUFA were not sequestered in LD, but the mechanisms of how PMP70 regulate LD abundance need further investigation.

Secondly, RNA-seq and flow cytometry analyses revealed that PMP70 depletion in HCT116L cells induced cell cycle arrest, particularly with a prolonged S phase. A more recent study demonstrated that arrested cells are more susceptible to LPO due to alterations in lipid composition of plasma membrane, which are regulated by MBOAT1 and EMP2 [44]. This raises the possibility that PMP70 depletion inhibited cell cycle progression, leading to decreased expression of MBOAT1 and EMP2, and consequently enhanced LPO. However, it would be of greater interest in the future to explore how PMP70 directly affects lipid composition, given its role in lipid metabolism. Furthermore, investigating the mechanisms by which PMP70 influences cell cycle progression is also warranted.

In summary, our study highlights the pivotal role of peroxisomes and PMP70 in maintaining chemoresistance in CRC through the regulation of LPO balance. An aberrant metabolic profile of PMP70-depleted cells was also observed, characterized by excessive LPO, loss of LDs and cell cycle arrest (Fig. 7E). Targeting PMP70 presents a promising therapeutic strategy to overcome LOHP resistance and improve treatment outcomes in CRC.

## Supplementary information

Supplementary Materials

Original data 1

## Data Availability

GSE106584 can be acquired from the Gene Expression Omnibus (GEO) datasets (https://www.ncbi.nlm.nih.gov/geo/). The data supporting the findings of this study are available from the corresponding author upon reasonable request.
