# Supplementary material for: Peroxisomal membrane protein PMP70 confers drug resistance in colorectal cancer
Source: Cell Death Dis. 2025 Apr 14;16(1):293. doi: 10.1038/s41419-025-07572-6 (PMC11997137; doi:10.1038/s41419-025-07572-6)
Supplement: Supplementary file 1 — Supplementary Materials [file 41419_2025_7572_MOESM1_ESM.pdf]

Supplementary Methods

RNAi and plasmid transfection

Supplementary Table 2 contains sequence or order information of siRNA. HA-Ub was purchased from Addgene (#18712). To construct mCherry-SKL, SKL sequence was inserted in the pmCherry-C1 backbone. To monitor the pexophagy system, we inserted an SKL sequence at the C-terminus of dimeric Keima-Red (dKeima), which was obtained from MBL (AM-V0101M). We constructed dKeima-SKL by digesting dimeric Keima with SKL insertion (dKeima-SKL) and ligating its fragments into similar sites in the pcDNA 3.1/MYC vector (Invitrogen, V85520).

CRISPR-Cas9 gene editing

The LentiCRISPR V2 vector was used to clone sgRNAs. Lentiviruses were generated from the sgRNA constructs in 293FT packaging cells using lipofectamine 3000 as a transfection reagent. The infected cells were selected with puromycin (2 μg/mL) starting 48 hours post-infection and propagated for further single-cell cloning. Transfected cells were sorted into 96-well plates at a density of one cell per well and grown for seven days until they became single-cell clones. The sequences of sgRNA are described: sgPMP70 #1: CTCGAACATATTGTGATGTT; sgPMP70 #2: ACCACCATTACAGAACAATG; sgPMP70 #3: AAAATGGGACACTAATTGAA; sgCtrl: GTCCACCCTTATCTAGGCTA.

Cell viability assay

Cell viability was assessed using Cell Counting Kit-8 (CCK-8, Dojindo, CK04) according to the manufacturer's protocols. Cells were seeded at a density of 5000 cells per well in 96-well plates and treated with LOHP or RSL3 for 48 hours. After treatment, fresh medium containing 10 μL of CCK-8 solution was added to each well, followed by incubation for 60–90 minutes. The absorbance at 450 nm was measured using a microplate reader (BMG CLARIOstar Plus), which reflects the number of viable cells.

Immunofluorescence

The cells were cultured in a confocal dish and then washed twice with 1 ml of cold 1 × PBS. After that, they were fixed in 4% paraformaldehyde (PFA) for 20 minutes and washed three times with 1 × PBS for five minutes at room temperature (RT). To permeabilize the cell membranes, we used 0.2% Triton X-100 for ten minutes in 1 × PBS, followed by washing as mentioned above. Next, we blocked the slides with 10% BSA/1 × PBS for one hour at RT and incubated them overnight at 4 °C in a humid chamber with primary antibodies (PMP70 and LC3) diluted to a ratio of 1:100 in 2% BSA/1 × PBS. After washing three times with 1 × PBS for five minutes each time at RT, we incubated the cells with secondary antibodies (rabbit 594 and/or mouse 488) diluted to ratio of 1:200 respectively for one hour at RT. The cells were then washed again three times using the same method as before and mounted using Antifade Mounting Medium containing Hoechst 33342 (Beyotime Biotechnology, China). Finally, we stored the dishes under dark conditions (4 °C) until viewing them under a Leica TCS SP8 MP Multiphoton Microscope.

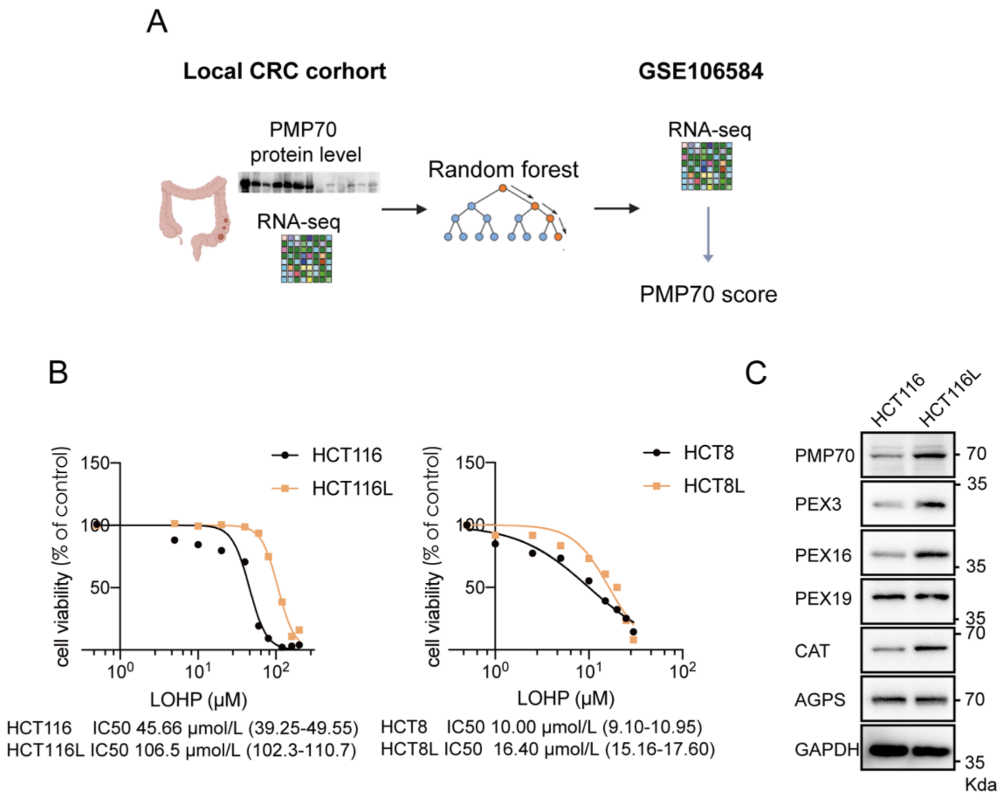

Figure S1 (A) Diagram showing the strategy for defining PMP70 score to predict peroxisome abundance or PMP70 protein level in GSE106584. (B) Survival curves for parental and LOHP-tolerant CRC cells (Left: HCT116 and HCT116L; Right: HCT8 and HCT8L cells) in response to LOHP. LOHP IC 50 values are shown. (C) Western blotting analysis of several proteins in the peroxisome pathway (PMP70, PEX3, PEX16, PEX19, Catalase and AGPS).

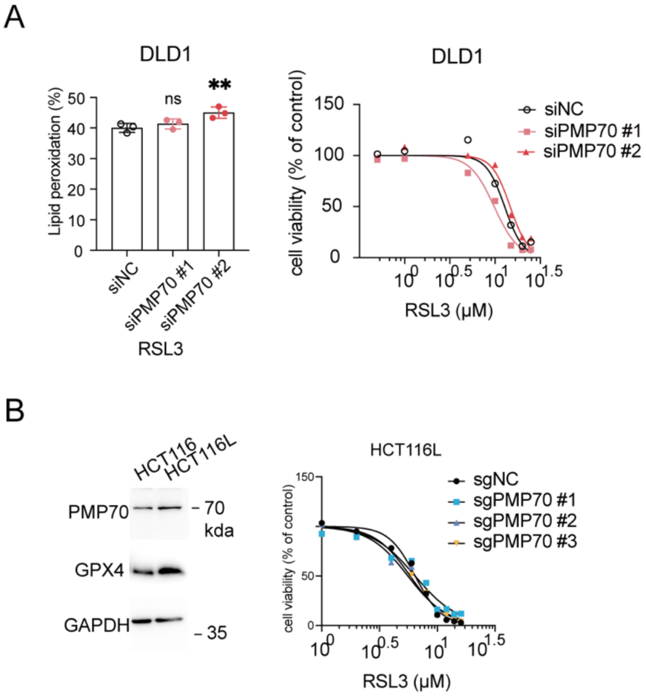

Figure S2 Down-regulation of PMP70 did not sensitize cells to RSL3-induced LPO and cell death.

(A) Lipid peroxidation levels and survival curves of DLD1 cells transfected with siPMP70 or control siRNA in response to RSL3 (7.5 μM). (B) Western blot analysis showing elevated PMP70 and GPX4 levels in HCT116L cells. Survival curves of HCT116L cells transfected with siPMP70 or control siRNA in response to RSL3. *P < 0.05, **P < 0.01, ***P < 0.001.

Supplementary Table 1. Clinicopathological characteristics of colorectal cancer patient samples.

Variable	Total cohort (n=36)	Variable	Total cohort (n=36)
**Age**		**Nerve invasion**	
≤ 60y	10	positive	5
>60y	26	negative	9
**Gender**		**Differentiation**	
male	20	Poor	2
female	16	Moderate and well	18
**T stage**		**MSI status**	
1-2	9	MSI	2
3-4	27	MSS	34
**Lymph node infiltration**		**Location**	
positive	19	Right	6
negative	17	Left, and rectum	30

Supplementary Table 2. Sequences of siRNAs.

**Gene**	**Number**	**Sequence**
PMP70	#1	GGGCGUGAAAUGACUAGAUTT
PMP70	#2	TCCGAGTAAGGCTCACTAA
GPX4	#1	GCTACAACGTCAAATTCGA
GPX4	#2	GTAACGAAGAGATCAAAGA

Supplementary Table 3. Details of antibodies.

**Antibodies**	**Source**	**Catalogue number**
PMP70	Abcam	Ab3421
PMP70	Santa	Sc-514728
CAT	CST	12980
GPX4	Abcam	Ab125066
AGPS	Abcam	Ab184186
PEX16	Proteintech	14816-1-AP
PEX19	Proteintech	14713-1-AP
CD133	CST	64326S
HA	Abclonal	AE105
LC3 I/II	CST	#4108
SOAT1	Abcam	Ab39327
DGAT1	Abcam	Ab178711
GAPDH	Proteintech	60004-1-Ig
