# Supplementary material for: Peroxisomal membrane protein PMP70 confers drug resistance in colorectal cancer
Source: Cell Death Dis. 2025 Apr 14;16(1):293. doi: 10.1038/s41419-025-07572-6 (PMC11997137; doi:10.1038/s41419-025-07572-6)

Figure 1A

Protein marker: Thermo #16626

PMP70

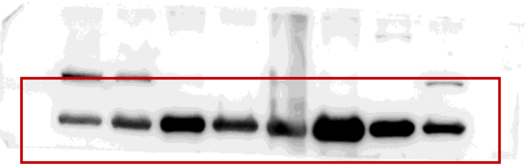

GAPDH

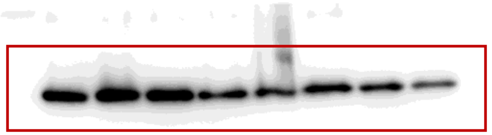

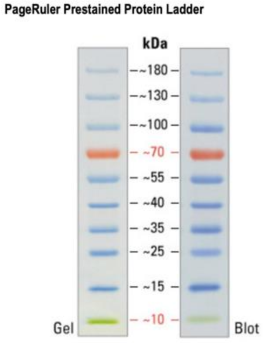

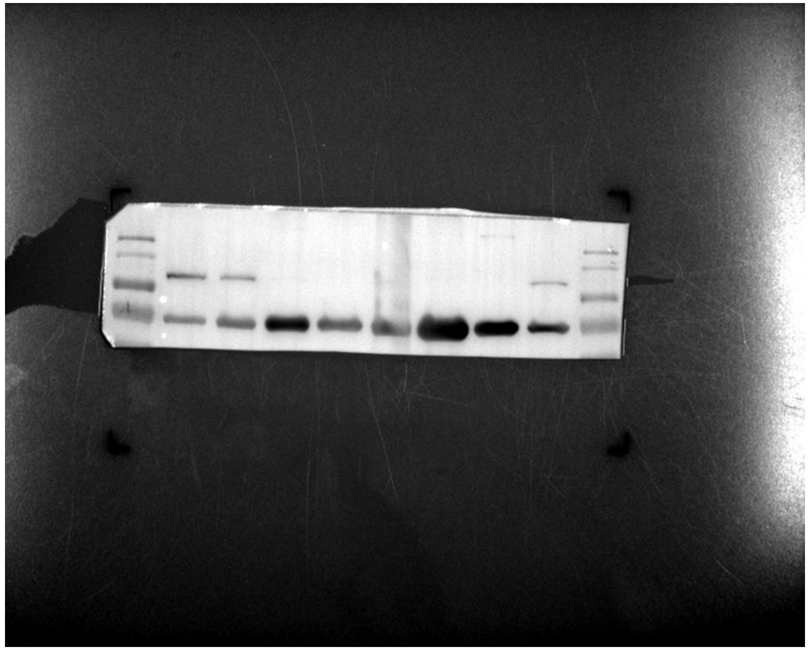

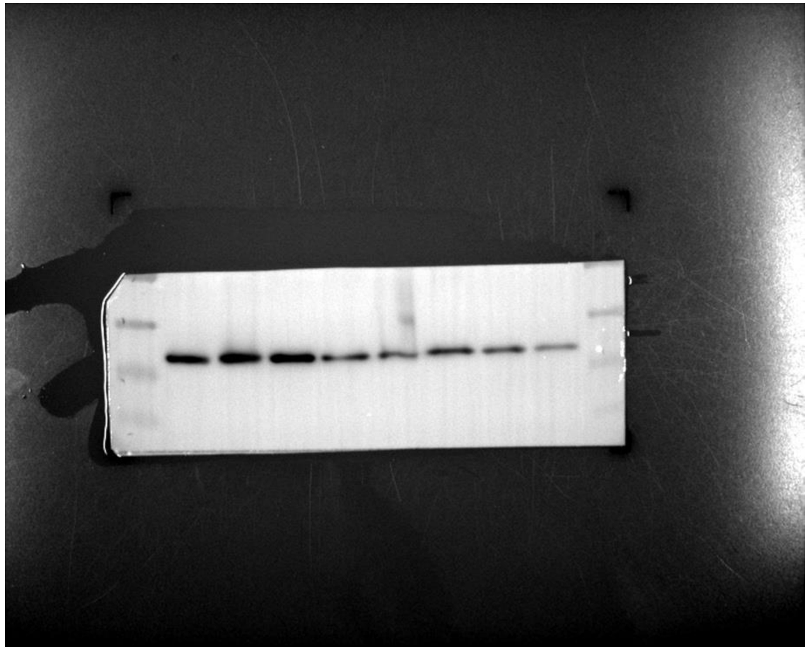

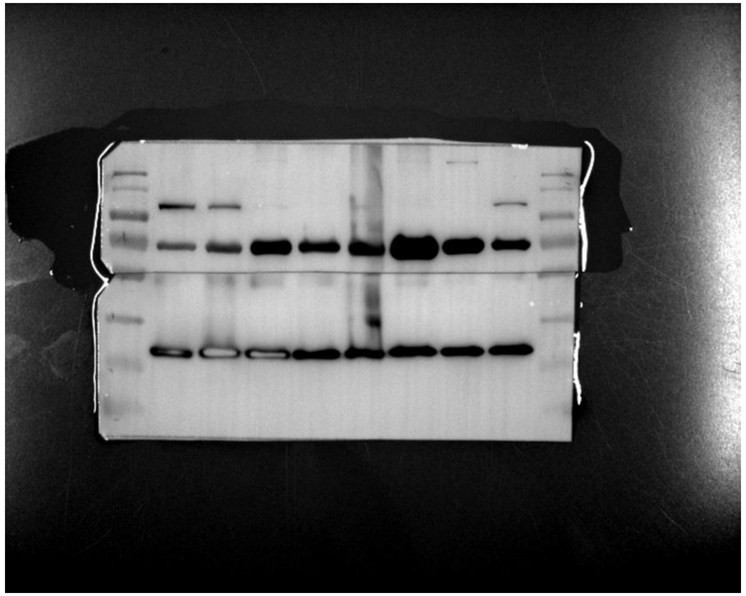

Figure 1F

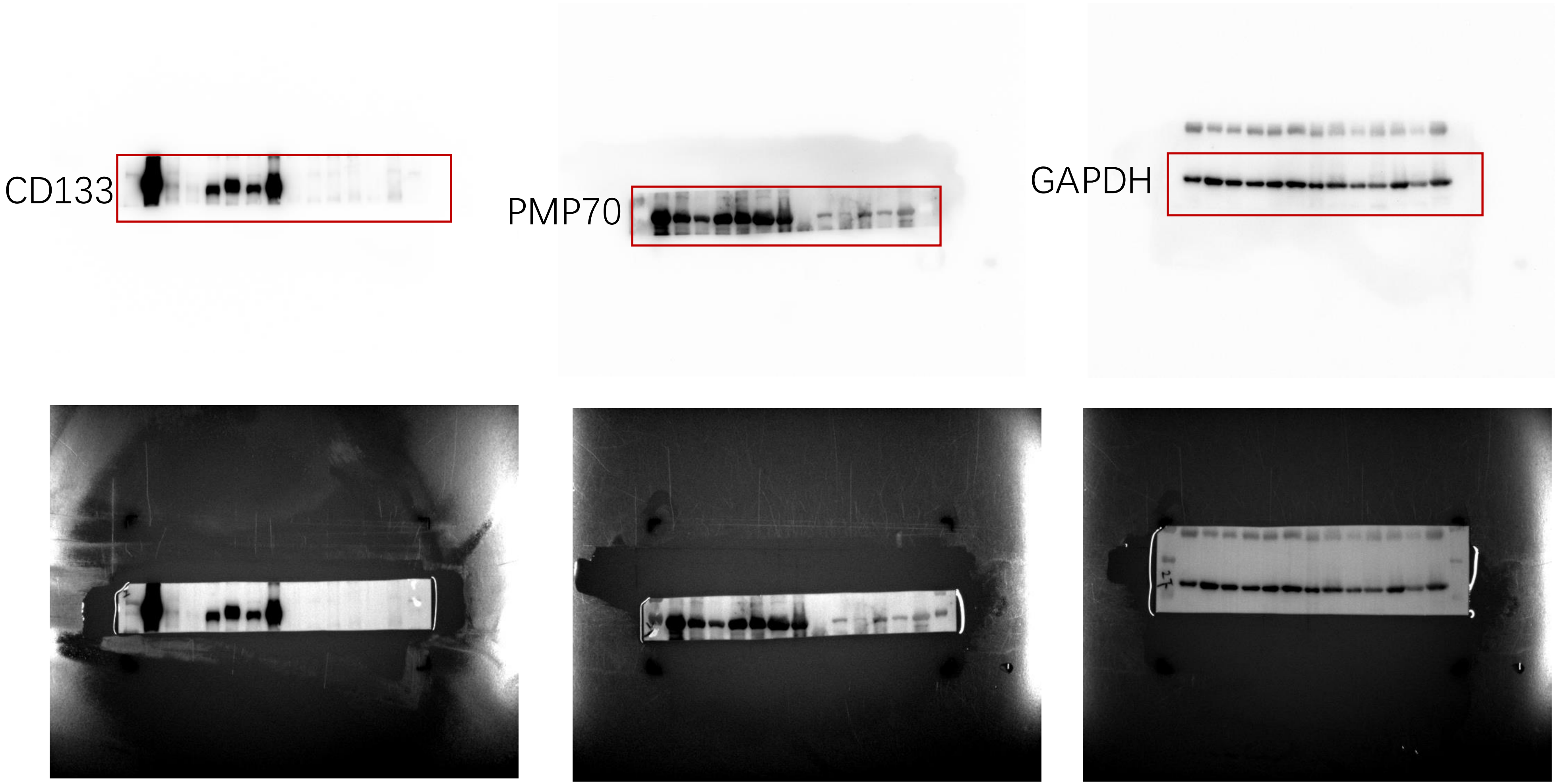

Figure 2I

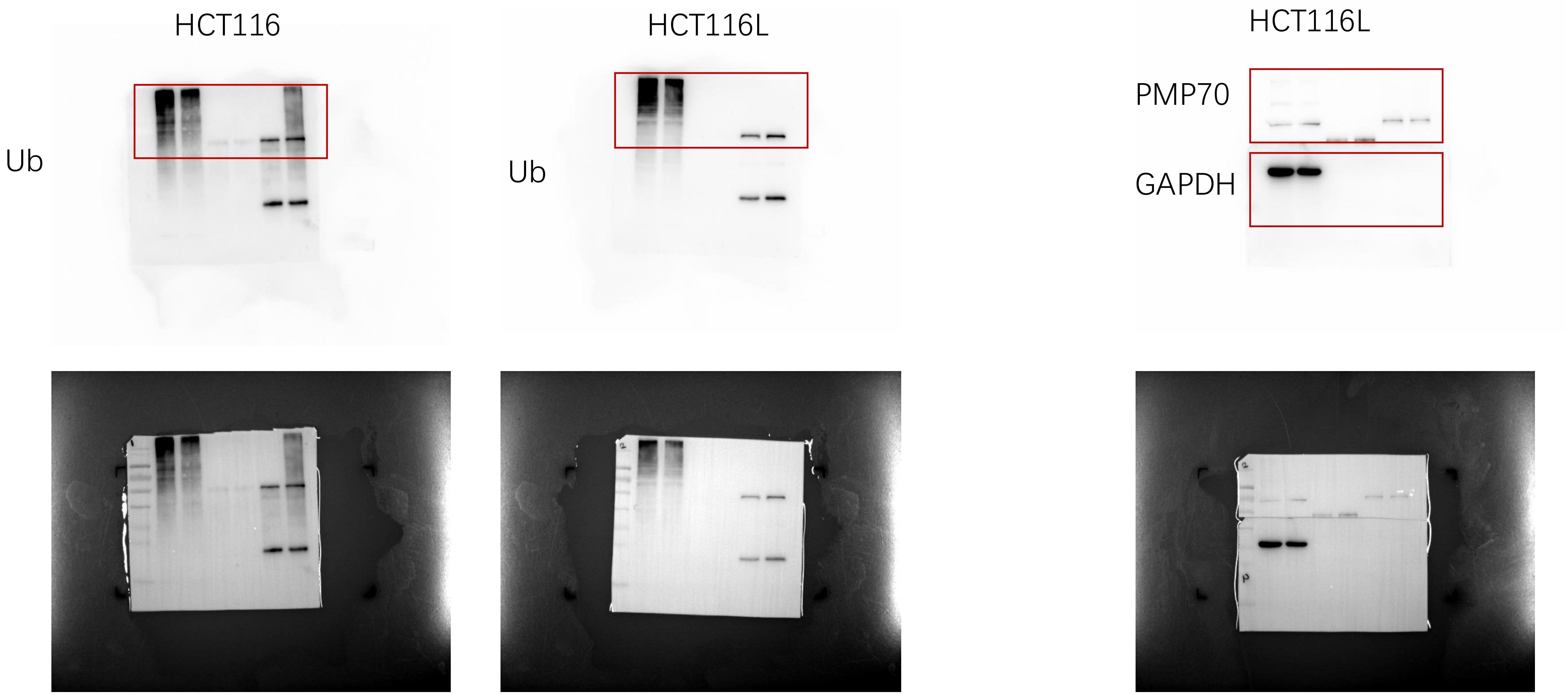

Figure 2I

HCT116

PMP70

GAPDH

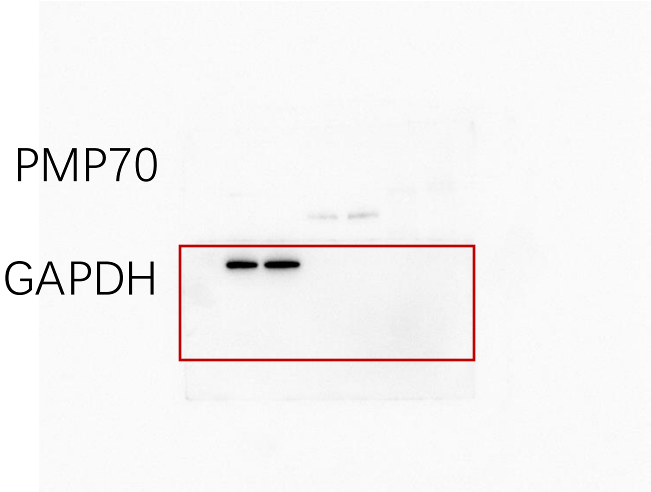

Exposure time:0.5s

HCT116: PMP70

HCT116L: PMP70

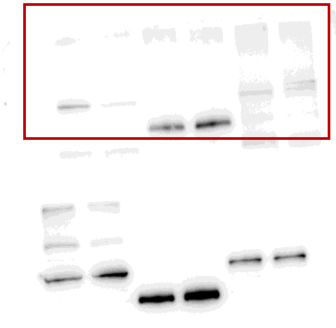

PMP70

GAPDH

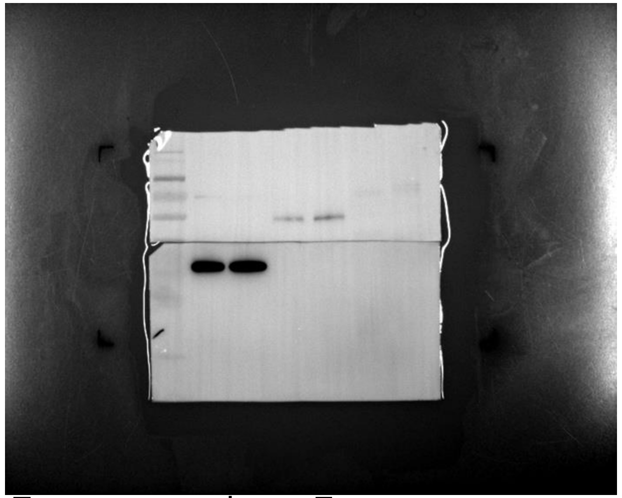

Exposure time:5s

HCT116: PMP70

HCT116L: PMP70

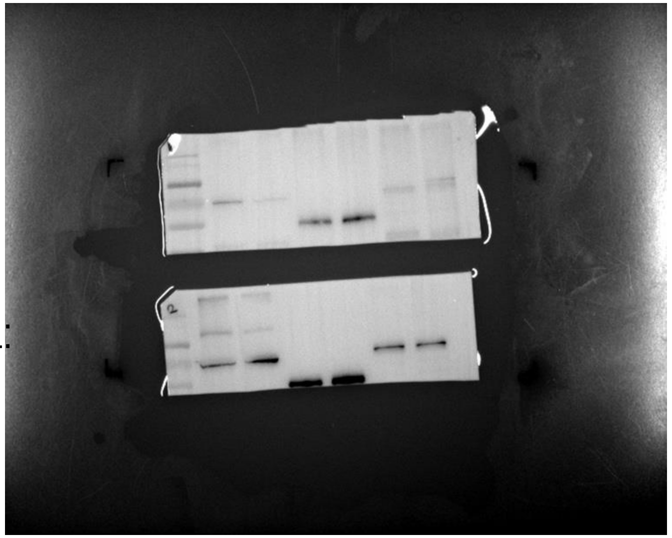

Figure 3C

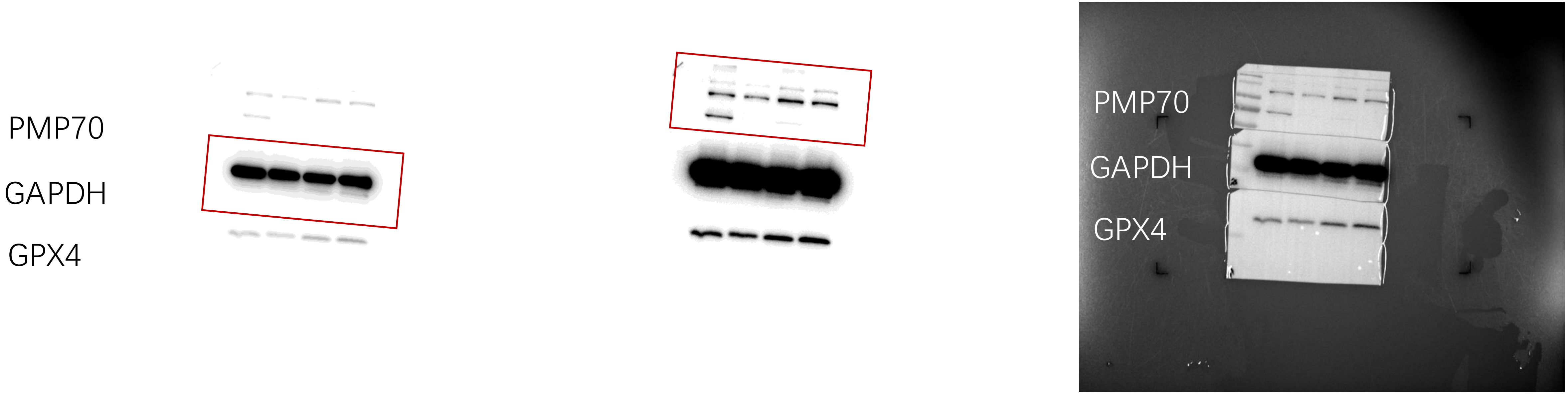

Exposure time:1s

Exposure time:5s

Figure 3C

CD133

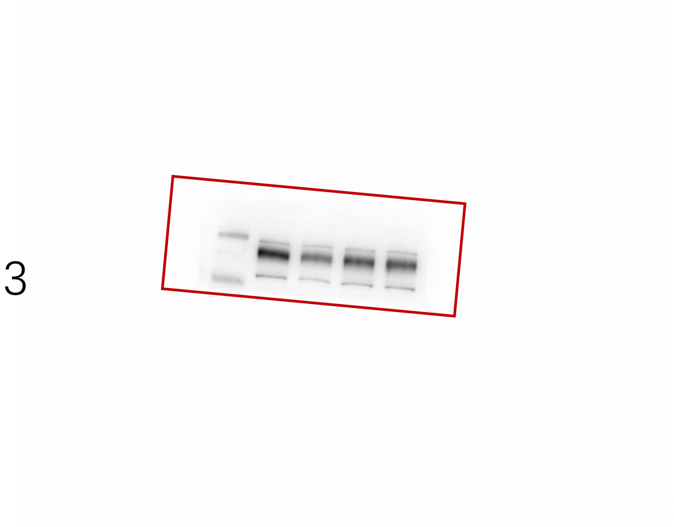

NANOG

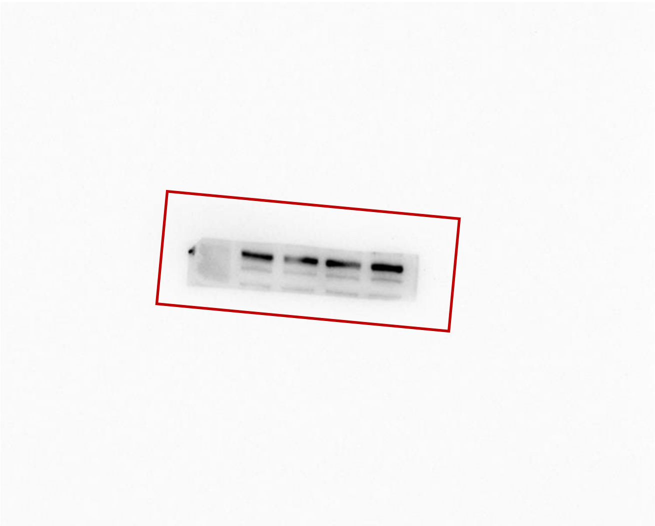

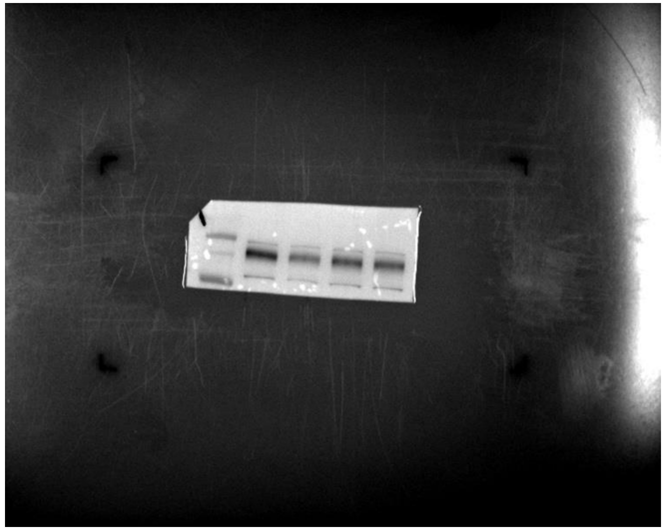

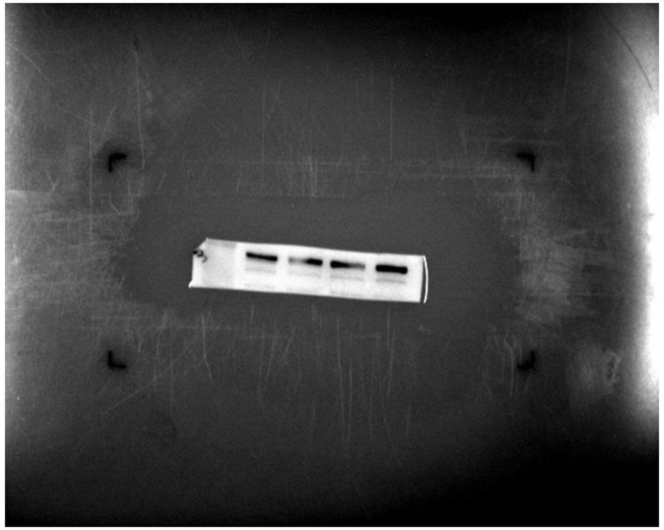

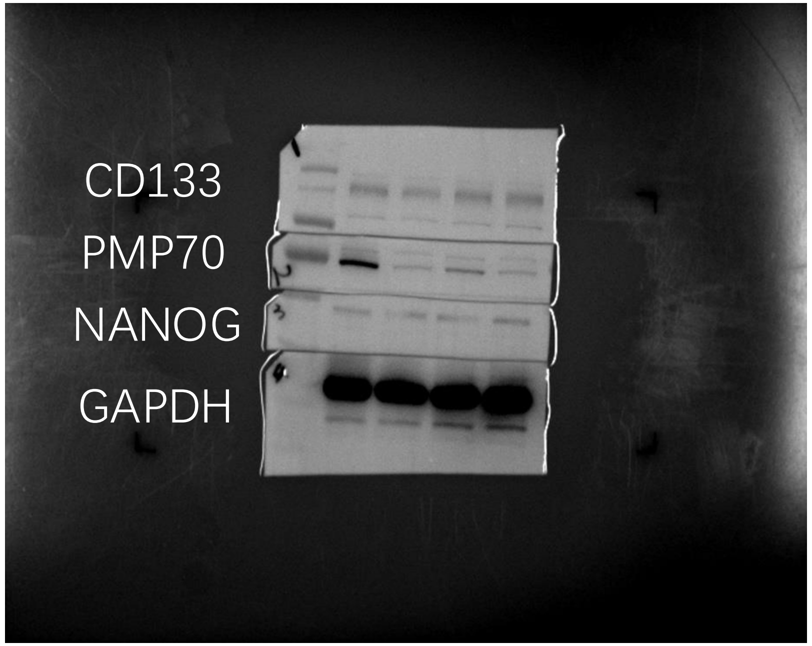

Figure 3C

CD44

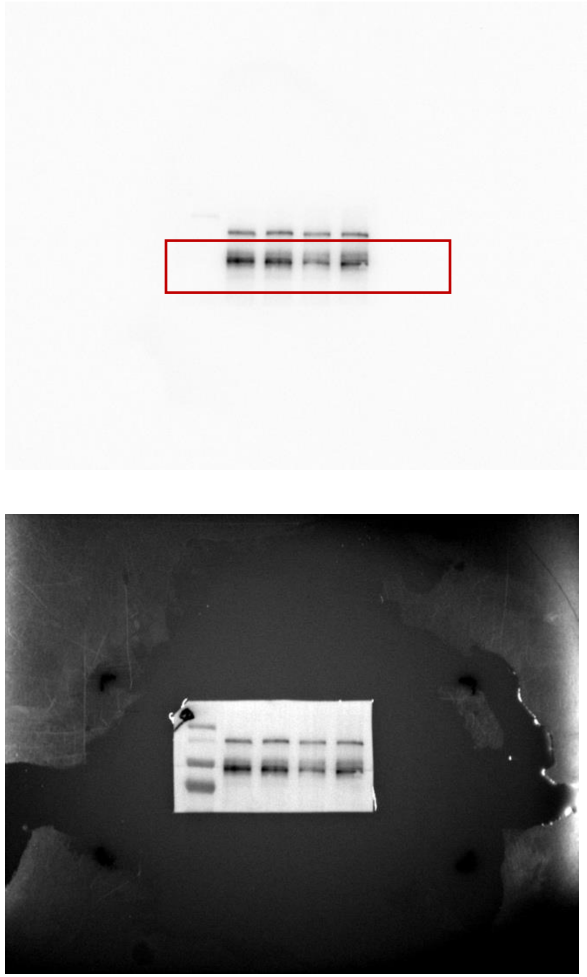

Figure 6F

SOAT1

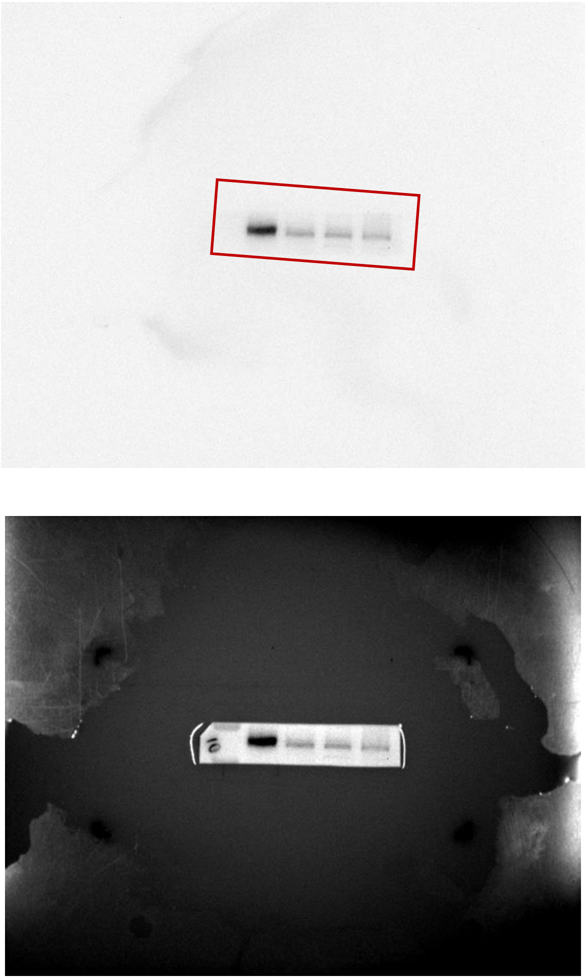

Figure 6F

GAPDH

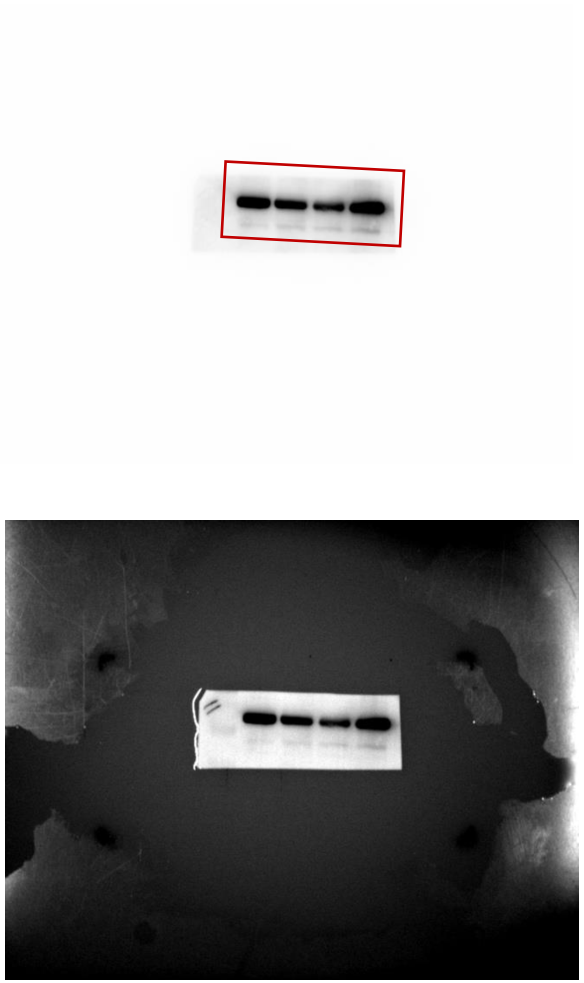

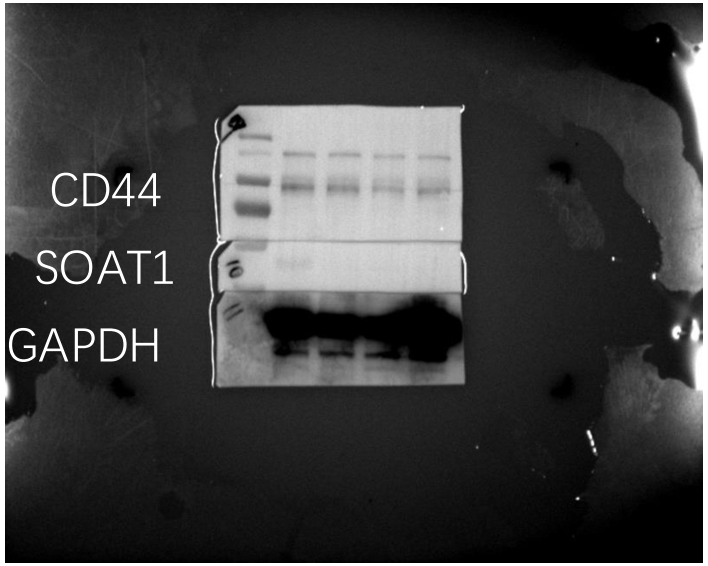

Figure 6F

DGAT1

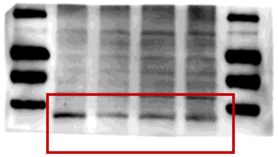

GAPDH

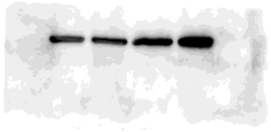

DGAT1

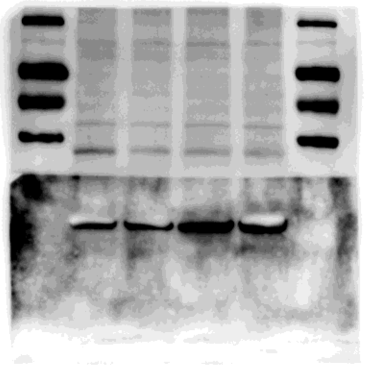

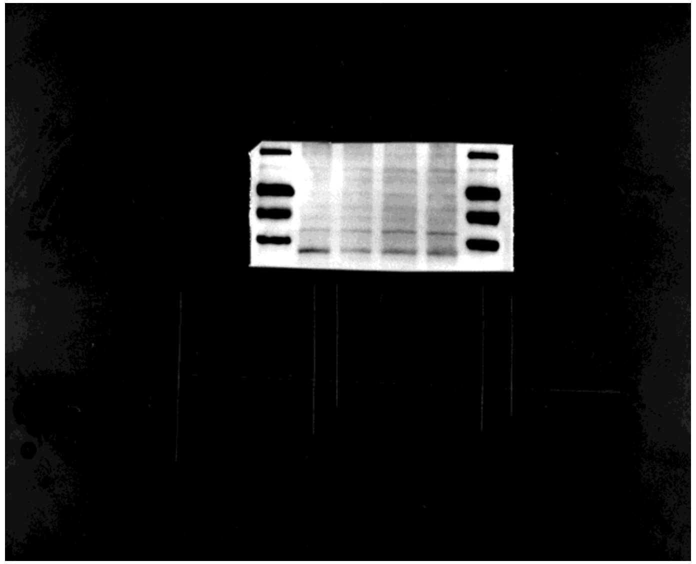

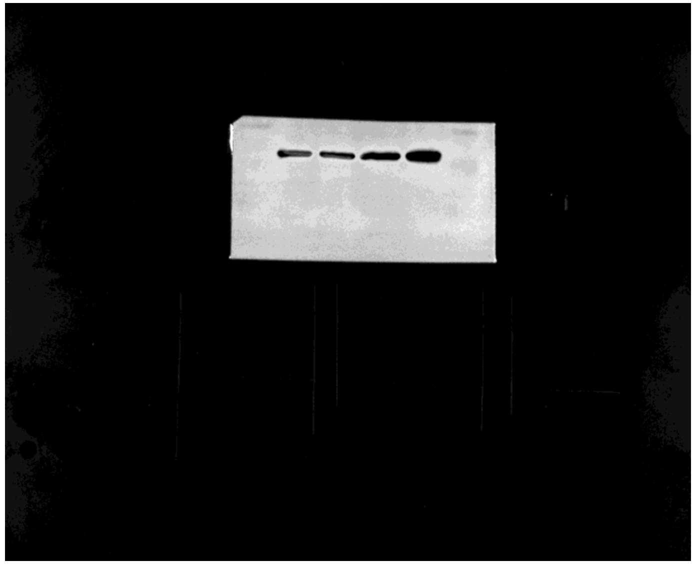

Figure 4F

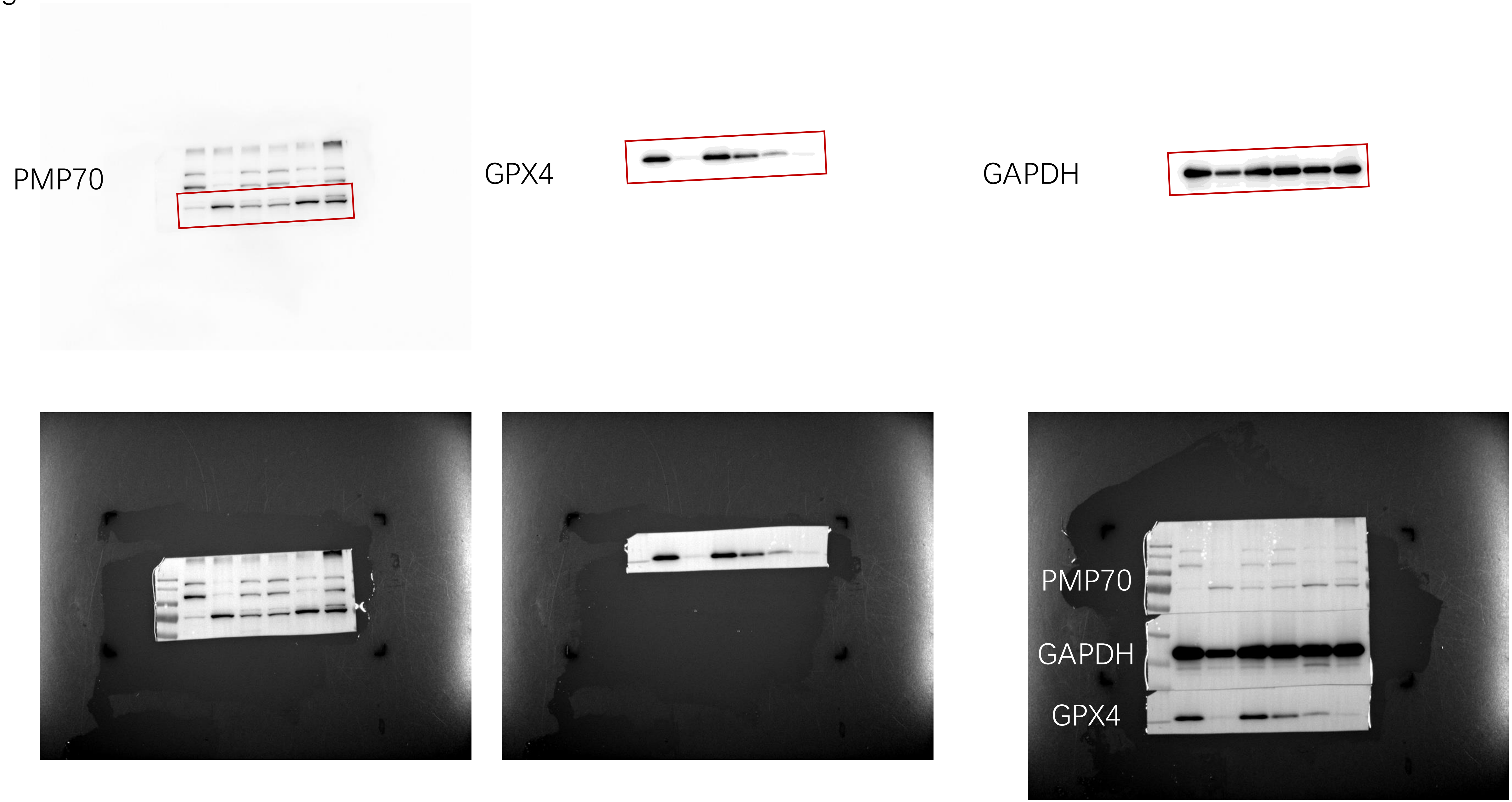

Figure S1C

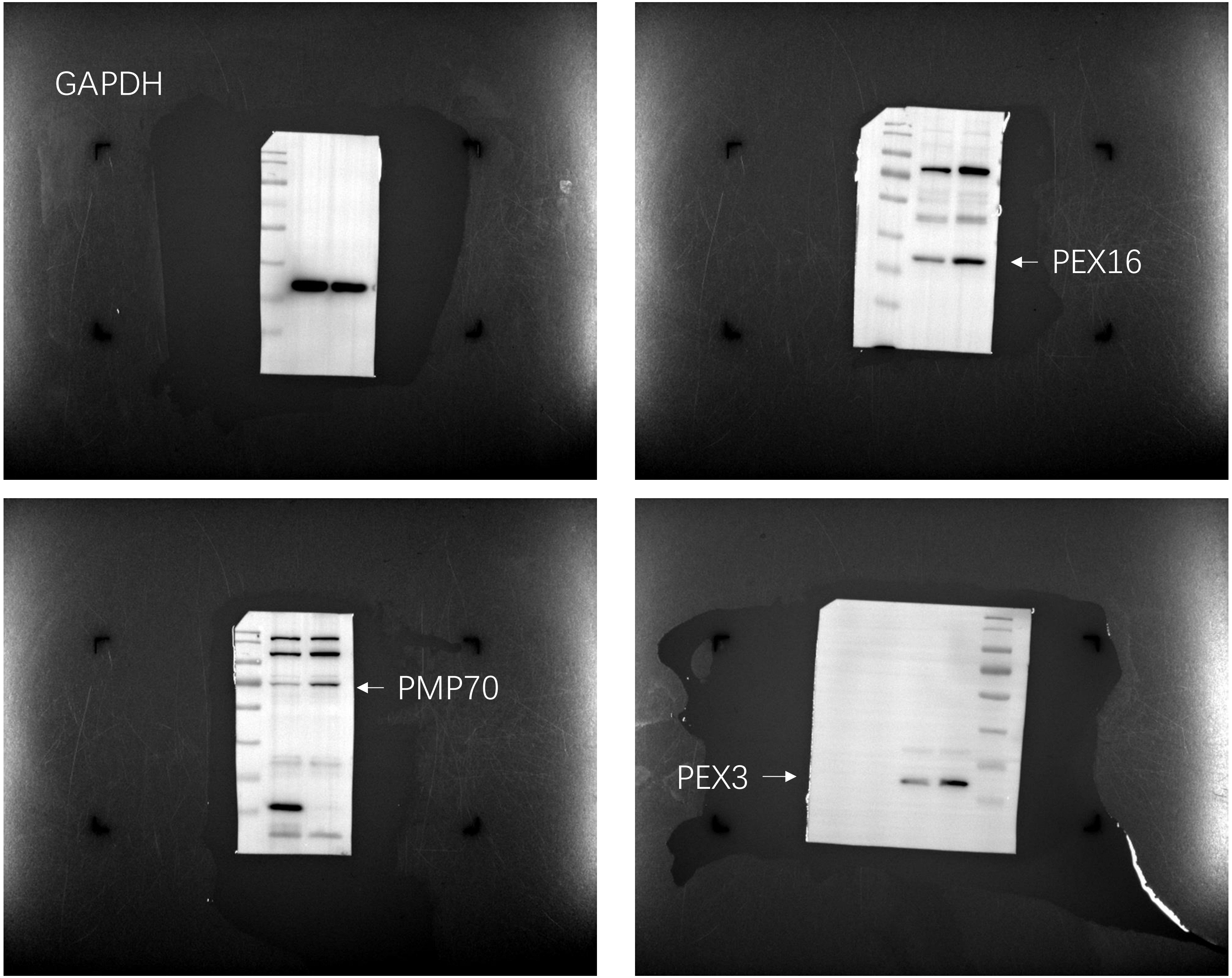

Figure S1C

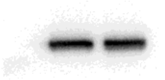

AGPS

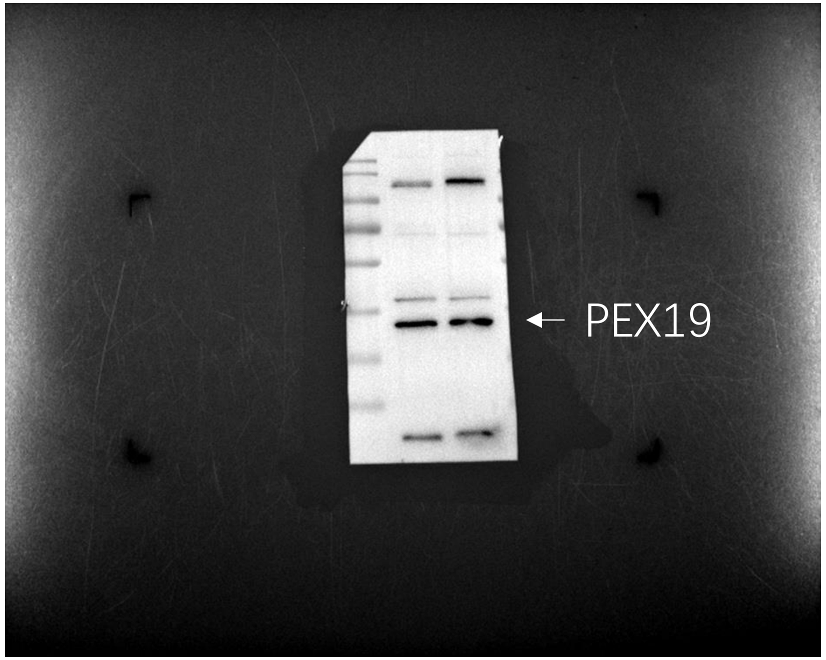

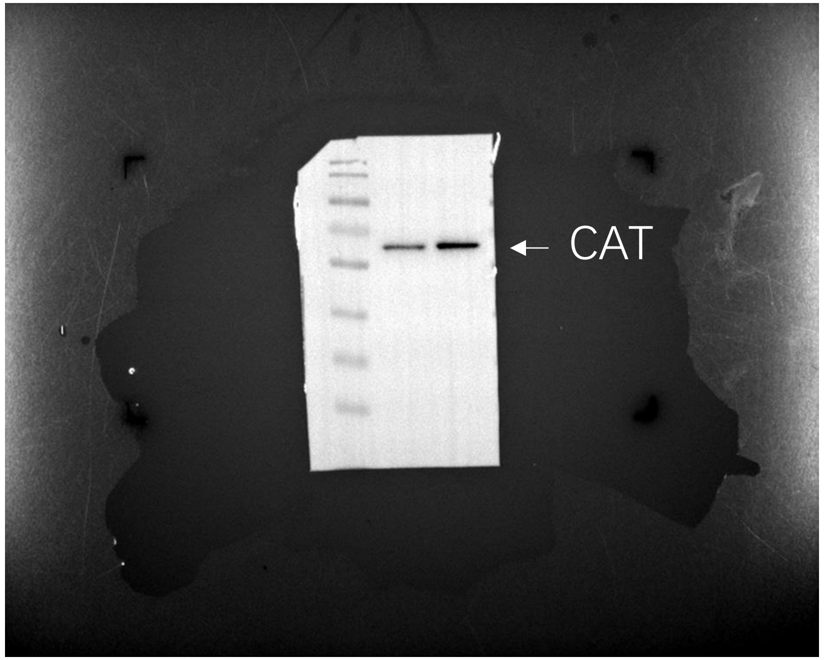

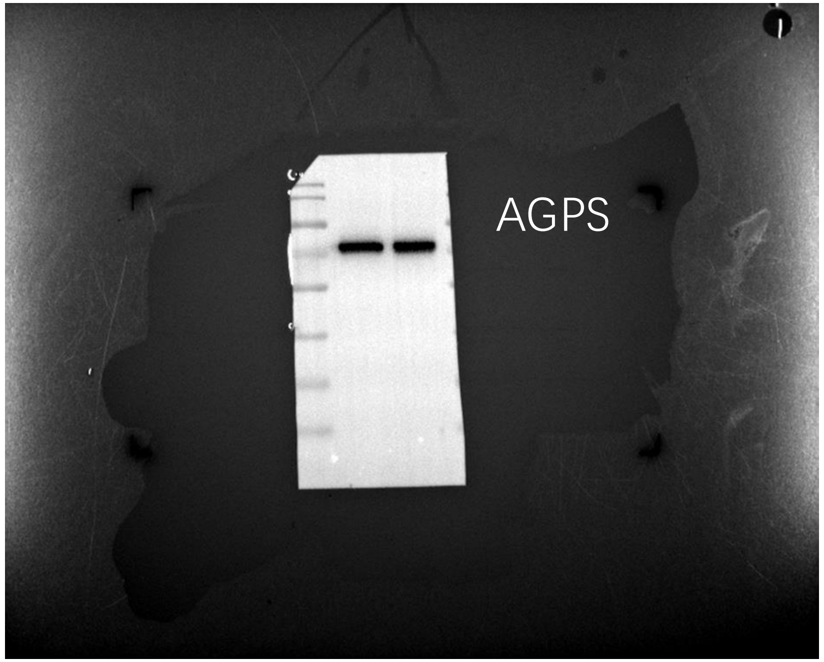

Supplement: Supplementary file 2 — Original data 1 [file 41419_2025_7572_MOESM2_ESM.pdf]
